# Incompatible pollen abortion and late-acting self-incompatibility in *Schima superba*

**DOI:** 10.1038/s41598-022-19946-3

**Published:** 2022-09-16

**Authors:** Rui Zhang, Hanbo Yang, Zhichun Zhou

**Affiliations:** 1 Research Institute of Subtropical Forestry, CAF, Zhejiang Provincial Key Laboratory of Tree Breeding, Daqiao Road 73, Fuyang, 311400 Zhejiang People’s Republic of China; 2 Sichuan Agriculture University, Chengdu, 610081 China

**Keywords:** Plant breeding, Plant development, Plant reproduction

## Abstract

In angiosperms, self-incompatibility (SI) is a common and widespread mechanism for plant prevention of inbreeding, and late-acting self-incompatibility (LSI) may be ancestral in the group. In this work, we studied *Schima superba*, a species in Theaceae that is a commercially important timer and fire-resistant tree, and revealed its LSI mechanism. Hormones, enzymes, transcriptomes, and proteins were compared between self-pollination (SP) and outcross pollination (OP) in the styles and ovaries from 0 to 120 h after pollination. The self-pollen tubes grew to the bottom of the style and entered the ovary within 48 h but failed to penetrate the ovule. Meanwhile, the hormone and peroxidase levels dramatically changed. Transcriptome and proteome analyses explored the molecular mechanisms of LSI and candidate genes related to LSI in *S. superba*. Overall, 586.71 million reads were obtained, and 79,642 (39.08%) unigenes were annotated. KEGG and GO analysis showed that there were 4531 differentially expressed genes (DEGs) and 82 differentially expressed proteins (DEPs) at 48 h in self- (SP) versus outcross pollination (OP). Among these, 160 DEGs and 33 DEPs were involved in pollen–pistil interactions. “Pollen–pistil interaction,” “signal recognition,” and “component of membrane” were downregulated in SP, whereas “cell wall and membrane biosynthetic process,” and “oxidoreductase activity” were upregulated. The DEGs involved with S-RNases and SCF during SP suggested that the LSI occurred at 48 h in the ovary and that the LSI in *S. superba* was under gametophyte control. Calcium ion increase and release, mitochondrial function loss, and ROS disruption further aggravated PCD progress and cell death. The LSI of *S. superba*, which happened 48 h after pollination, was a key time point. The incompatibility PT ceased growth in the ovary because of S-RNase recognition and PCD in this organ. This study highlights the LSI molecular mechanism in *S. superba* and provides a reference to other species in Theaceae.

## Introduction

In angiosperms, self-incompatibility (SI) is a common and widespread mechanism to reduce the risk of inbreeding depression, and it is usually under genetic control^1,2^. SI systems are either sporophytic SI (SSI) or gametophytic (GSI)^3,4^. SSI always occurs in the stigma, and the pollen-specific S-locus cysteine-rich protein (SCR) and stigma-specific S-receptor kinase (SRK) both control pollen tube (PT) growth; the SSI pattern’s novel species is in *Brassicaceae*^5,6^. In GSI systems, the S-RNase (the female determinant) and *S*-locus F-box protein (SLF/SFB, the male determinant) interact to mediate the self-pollen tube rejection response, and this pattern is most common in the styles of *Plantaginaceae*, *Solanaceae* and *Rosaceae*^7–9^. In *Papaveraceae*, another kind of GSI is caused by the Ca^2+^ signaling cascade, leading to apoptosis and the termination of the pollen tube (PT) growth^10^. Additionally, ovarian SI (OSI) or late-acting SI (LSI) occurs when the PT ceases growth in the ovary^11^. LSI, an ancestrally conserved mechanism of SI, exists widely among *Narcissus papyraceus*, *Clivia gardenia*, *Aloe maculate*, *Theobroma cacao*, and *Thryptomene calycina*^12–14^.

Since abortion could occur before or after zygote formation, in addition to a much more complicated mechanism in postzygotic SI, the molecular mechanisms of LSI are poorly understood^14–16^. LSI can be controlled by the SSI, GSI, or both^3^. Studies have shown that some growing molecular signals involved in PT attraction to the synergid cells and ovules affect self-PT growth differentially in LSI species^17–20^. For example, SCR/s-locus protein 11 (SP11), which is a synergid-derived cue (LURE) belonging to the supergene family CRPs in *Brassica* pollen, comprises a male signaling ligand determinant^21^. Growth of the self-pollination tube is halted in the style, whereas SCF and *S-RNase* genes expressed in *Camellia sinensis* show that LSI is under gametophytic control^22^. Sage predicted that the LSI system’s genetic basis is always gametophytic^11^. Diallel crosses with sibling progeny arrays suggest that multiple loci with multiple alleles control the LSI response^23,24^.

**Figure 1:**
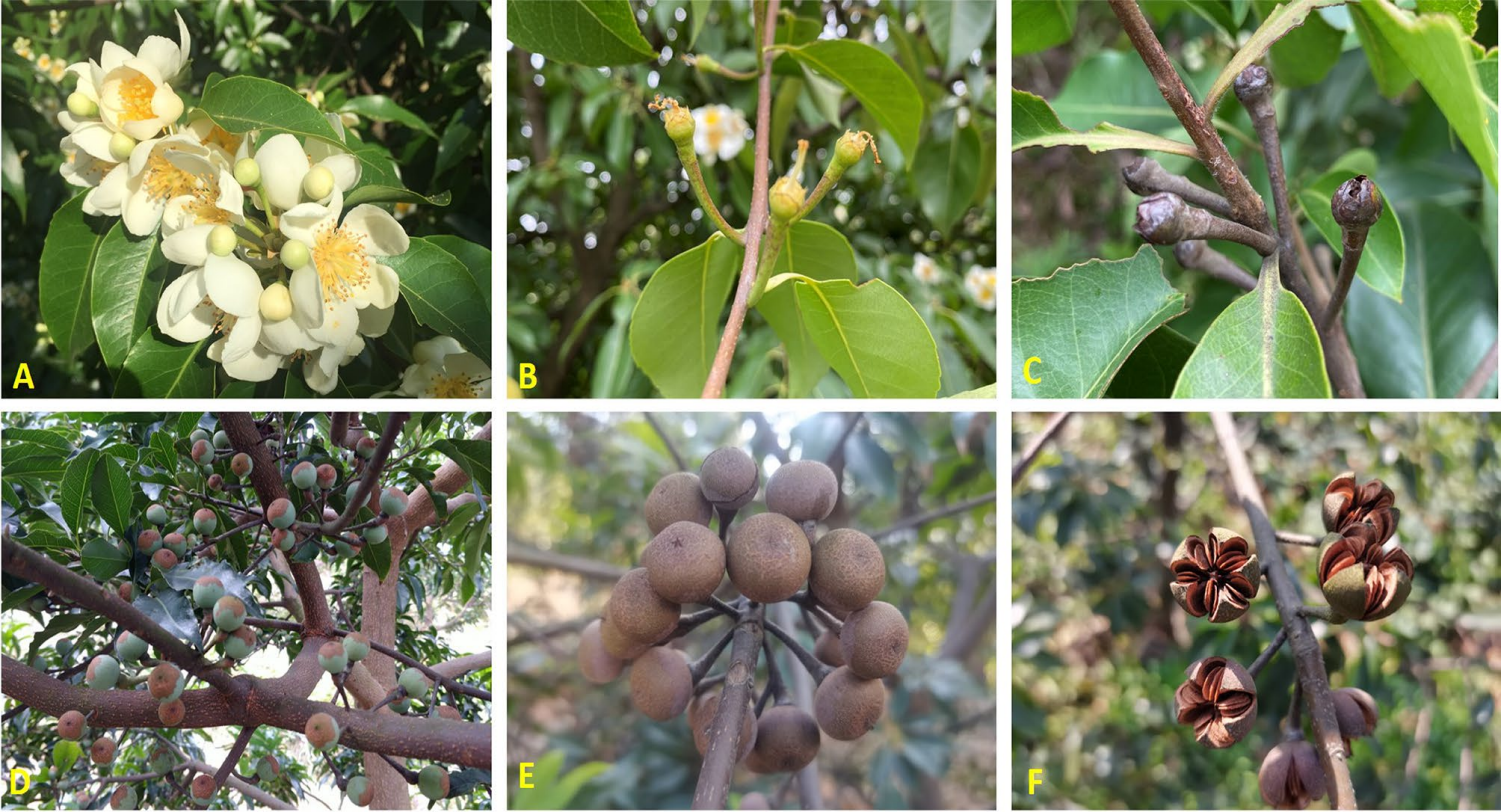
Photographs of flowers (**A**), 72 h fruits after pollination (**B**), 6-month fruits (**C**), 13-month fruits (**D**), 16-month fruits (**E**), and cracked fruits and seeds (**F**, 17 months) of *S. superba*.

**Figure 2:**
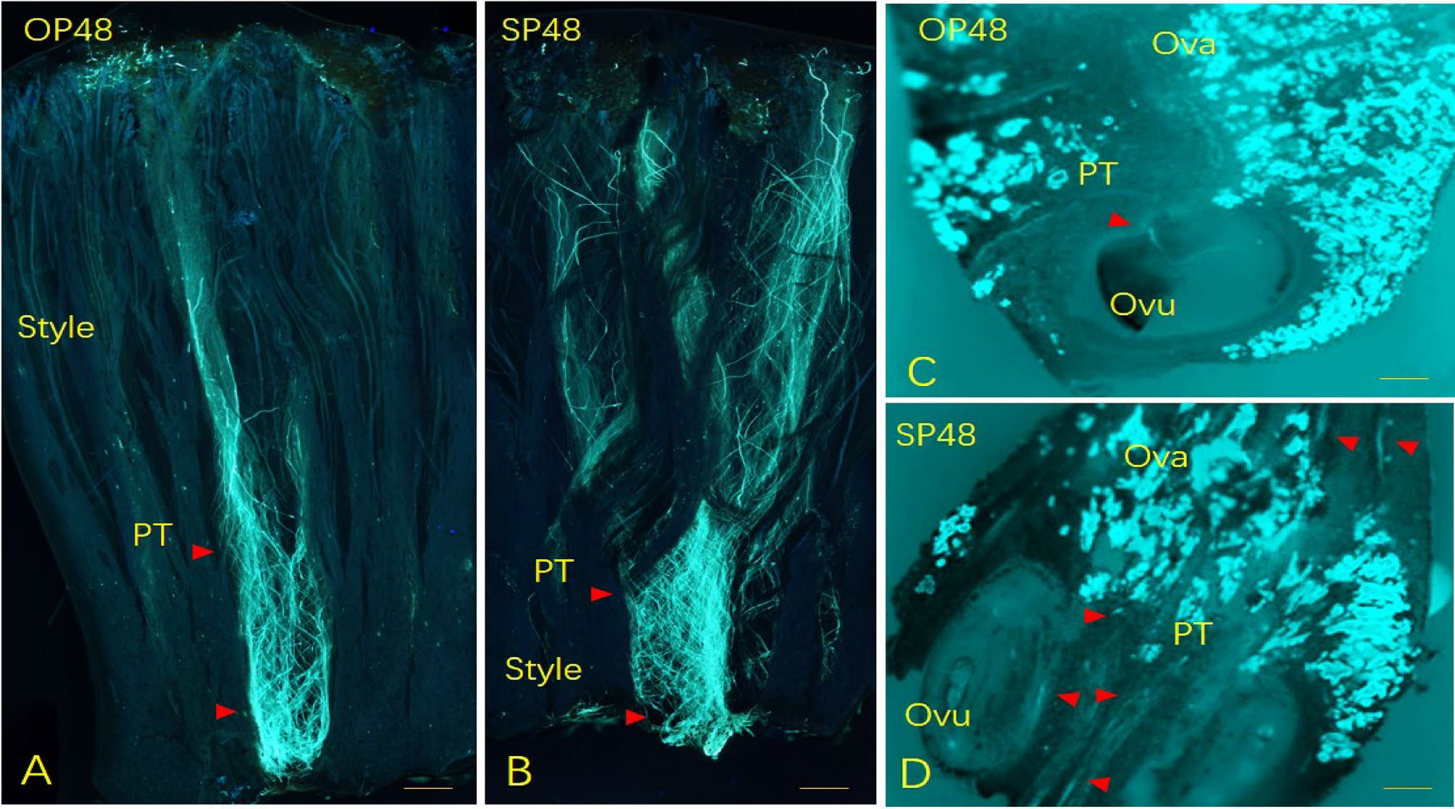
Pollen tubes growth of *S. superba* during SP and OP at 48 h in style and ovary. PTs during OP at 48 h in the style (**A**) and ovary (**C**), and they were during SP at 48 h in the style (**B**) and ovary (**D**). PTs were shown by red arrows. SEM resolution of 200 μm.

*Schima superba* is a woody plant in the tribe Schimeae in Theaceae, which is well-known for the genus *Camellia* in the Theeae tribe. These two tribes have phylogenetic similarities^25,26^. Currently, research on SI has made great progress, and several metabolic pathways are regulated by multiple genes in *C. sinensis*^22,27^. *Schima superba* is widely distributed in southern China, and its function is mainly for biological fireproofing and valuable timber^28,29^. Its fluorescence occurs from the middle of May to the middle of July, and its flowers are small to moderate (length_corolla_ = 31.57 mm) (Fig. 1). In our preliminary study, during outcross pollination (OP), the PTs grew rapidly, reached the bottom of style at 36 h and then penetrate the ovules at 48 h (Fig. 2A,C), however, during self-pollination (SP), the PTs grew slowly from 2 to 36 h after pollination, and then they entered in the ovary, passed the ovule without touching at 48 h (Fig. 2B,D)^30^.The fruit abortion rates were over 88% in the SP treatment, and seed yield was only 0.2–0.4%. Therefore, we deduced that *S. superba* is an LSI species. To clarify the LSI molecular mechanism of *S. superba*, we compared hormone levels, such as IAA, ABA, and ZT, between SP and OP in styles and ovaries from 0 to 120 h after pollination. Through physiological results, combined with early anatomical section observation, we further confirmed the accurate time point of PT growth inhibition. Then, at this time point, we associated the transcriptome with proteome analysis to reveal the gene expression pattern, especially to determine whether SSI (S-locus protein), or GSI (SCF/S-RNase), or their combination control PT growth. The results explain the LSI mechanism in *S. superba* and refresh knowledge in Theaceae.

## Results

### Hormone and enzyme quantification in SP versus OP

**Figure 3:**
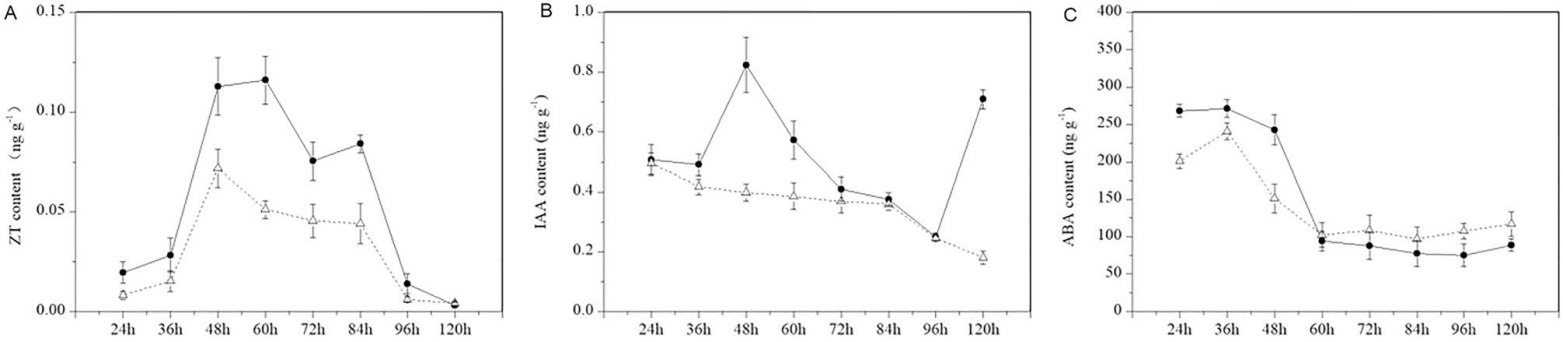
Hormone content in SP and OP. (**A**) Zeatin, ZT. (**B**) Auxin, IAA. (**C**) Abscisic acid, ABA.

To further confirm the PT growth inhibition time point, a quantitative analysis of hormone levels in SP and OP was revealed by HPLC–MS/MS. IAA and ZT biosynthesis were decreased in SP from 24 to 120 h (Fig. 3A,B); however, ABA increased after 60 h in SP (Fig. 3C). The changes in the hormone level showed that 48 h after pollination was a special turning point, in which IAA, ZT, and ABA were downregulated by 52, 36 and 38%, respectively, in SP ovaries compared to OP ovaries.

**Figure 4:**
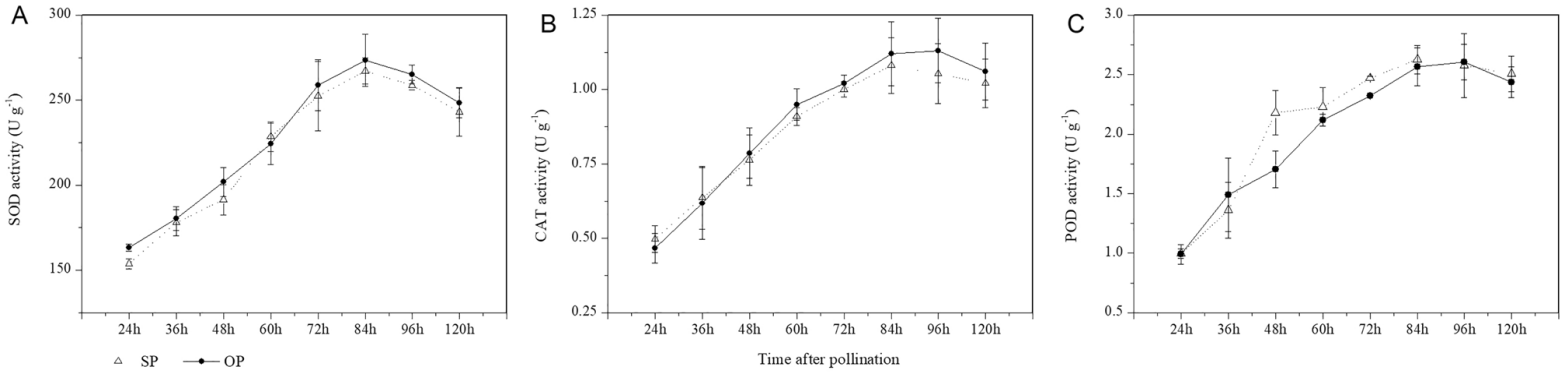
SOD (**A**), CAT (**B**) and POD (**C**) activities in SP and OP.

Changes in the antioxidant system were important for determining the time point of self-pollinated PT growth inhibition. CAT, POD, and SOD activity increased from 24 to 84 h in both SP and OP (Fig. 4A,B). Although the POD activity was high from 48 to 84 h in SP versus OP, the CAT and SOD activity was low, and the mean value was decreased by 2.3% and 2.7% in SP versus OP (Fig. 4).

### DEGs in self- and outcrossed ovaries

#### Illumina sequencing, de novo assembly, and functional annotation

To identify genes changed during PT growth in the ovary 48 h after OP and SP treatment in *S. superba*, six libraries (three replicates of OP and SP ovaries 48 h after pollination), were sequenced with the Illumina HiSeq 2000 platform. A total of 586.71 million bases of raw reads and 569 million clean reads (Q30 > 92.86%) were generated. In total, 85.36 Gb of nucleotides, 203,767 unigenes, and 79,642 (39.08%) annotated unigenes were generated (Table 1, Supplementary Table S1).

**Table 1:** Summary of functional annotation.

	Number	Percentage (%)
Nr Annotated	61,549	30.2
Nt Annotated	42,395	20.8
Swiss-Prot Annotated	44,414	21.79
KEGG Annotated	22,088	10.83
GO Annotated	45,663	22.4
Annotated in KOG/COG	21,329	10.46
All annotated unigenes	79,642	39.08
Total Unigenes	203,767	100

**Figure 5:**
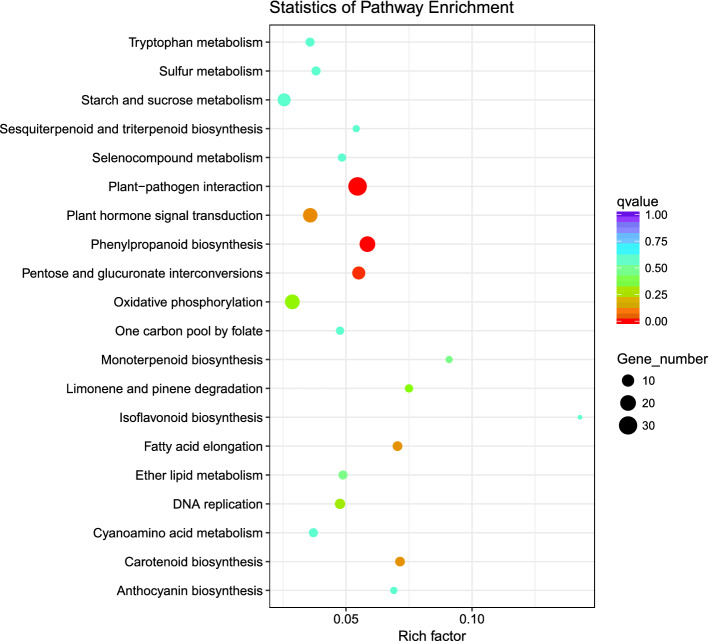
Statistic of pathway enrichment in KEGG of the DEG of SP versus OP.

A fold change threshold of ≥ 2.00 and *p* value ≤ 0.05 were used to identify differentially expressed genes (DEGs). There were 4531 DEGs identified in the SP versus OP ovaries, of which 2078 were upregulated and 2453 were downregulated (Supplementary Table S2 and Supplementary Figure S1). The gene ontology (GO) annotation assigned these DEGs to 2453 GO terms and classified them into cellular components (CC: 14%), molecular functions (MF: 24%), and biological processes (BP: 62%) (Supplementary Table S3). Furthermore, 62 putative transcription factor families were identified, and DEGs containing AP2-EREBP were the most abundant (9), followed by bHLH (8), MYB (8), and ABI3VP1 (6) (Supplementary Figure S2). In total, 338 DEGs were categorized into 84 KEGG pathways, 11 of which were significantly enriched (corrected P value ≤ 0.05): phenylpropanoid biosynthesis (5.8%), plant–pathogen interaction (5.5%), Pentose and glucuronate interconversions (5.5%), plant hormone signal transduction (3.6%), Carotenoid biosynthesis (7.1%), Fatty acid elongation (7.0%), DNA replication (4.8%), Oxidative phosphorylation (2.9%), Limonene and pinene degradation (7.5%), ether lipid metabolism (4.9%), and monoterpenoid biosynthesis (9.1%) (Fig. 5). In addition, “ubiquitin-mediated proteolysis,” “PCD,” and “calcium ion signaling” pathways related to SI were identified as upregulated in SP.

### Specifically or preferentially expressed genes involved in SP and OP

To identify differentially expressed genes associated with LSI in *S. superba*, we compared up- and downregulated DEGs in GO enrichment. Of the 4531 DEGs that were annotated into 2453 GO terms, the upregulated categories were related to “cell wall and membrane biosynthetic process” (13), “cuticle development” (1), and “glycolipid metabolic process” (7) in BP, “host intracellular organelle and membrane” (10) in CC, and “oxidoreductase activity” (16), “ribonuclease T2 activity” (3), and “binding” (21) in MF. However, “pollen–pistil interaction” (9), “recognition of pollen” (9), “response to stress” (8), and “glucose metabolic process” (7) in BP, “signal recognition” (2) and “component of membrane” (32) in CC, “transferase activity” (28), “cellulose synthase activity” (7), and “pheromone activity” (3) in MF were downregulated in SP (S48 vs O48) (Supplementary Table S3 and Supplementary Figure S3).

A total of 35 DEGs were annotated in the top 10 KEGG pathways and the top 15 of GO enrichment analysis and were related to ADP binding (5, K13459|K02133), cell wall (7, K13457|K01051), pollination (1, K15397), oxidoreductase activity (13, K09755|K00430|K09843|K00517|K02256), auxin (5, K14488), oligosaccharide (1, K01213), and phosphors signal (3, K14491|K14492|K14500) (Table 2). Furthermore, sesquiterpenoid and triterpenoid biosynthesis (oxidoreductase activity, c119780_g1), glycan biosynthesis (membrane lipid biosynthetic process, c89162_g1), and some immunity-related pathways were upregulated, and some pathways related to amino acid metabolism (Cysteine, c113534_g5|c109638_g2|c123367_g5; Histidine, c109070_g5|c109070_g2; Tyrosine, c118577_g1) and zeatin biosynthesis (c116756_g1) were downregulated in relation to LSI in *S. superba*.

**Table 2:** DEGs annotated in the top 10 KEGG and top 15 GO pathways.

KEGG Term	*p* value	UniGenes	KO	Gene name
Plant–pathogen interaction	0.000	C120244_g1|c126261_g1|c77700_g2|c98405_g1|c111738_g3	K13457|K13459|K13459|K13459|K13459	RPM1, RPS3|RPS2|RPS2|RPS2|RPS2|
Phenylpropanoid biosynthesis	0.000	c113063_g3|c99127_g2|c109745_g1|c112534_g4|c87454_g1|c76665_g1| c127124_g5|c87454_g2| c49580_g1	K09755|K00430|K00430|K00430|K00430|K00430|K00430|K00430| K00430	CYP84A, F5H| E1.11.1.7|E1.11.1.7| E1.11.1.7|E1.11.1.7|E1.11.1.7|E1.11.1.7|E1.11.1.7|E1.11.1.7
Pentose and glucuronate interconversions	0.015	c89170_g1|c125667_g1|c87333_g2|c63625_g1|c88144_g3|c88144_g2|c88144_g1	K01051|K01213|K01051|K01051|K01051|K01051|K01051|	E3.1.1.11|E3.2.1.67|E3.1.1.11|E3.1.1.11|E3.1.1.11|E3.1.1.11|E3.1.1.11|
Plant hormone signal transduction	0.115	c119184_g1|c19937_g1|c113234_g5|c105124_g2| c98108_g1|c106334_g2|c104870_g1|c113246_g2	K14488|K14491|K14492|K14500|K14488|K14488|K14488|K14488	SAUR|ARR-B|ARR-A|BSK| SAUR|SAUR|SAUR|IAA|
Carotenoid biosynthesis	0.128	c98441_g1|c115612_g1|	K09843|K09843	E1.14.13.93|E1.14.13.93
Fatty acid elongation	0.128	c84875_g1	K15397	KCS
Oxidative phosphorylation	0.314	c26116_g1| c107429_g1	K02256|K02133	COX1| ATPeF1B, ATP5B, ATP2
Limonene and pinene degradation	0.332	c95477_g1	K00517	E1.14.-.-

Of 62 TFs expressed specifically in the self-pollinated ovaries of *S. superba*, one MYB (c123606_g4) was significantly upregulated; GRAS(c64954_g2), BBR/BPC (c102379_g1), HSF (c117518_g3), and MBF1 (c110410_g3) were specifically upregulated, and FAR1(c107861_g4|c55999_g2|c112693_g3), C2H2 (c148204_g1| c96967_g1), HB (c104947_g1), zf-HD (c95393_g1| c87920_g1), G2-like (c29149_g1), PLATZ (c118044_g7), RWP-RK (c116010_g1), TCP (c104077_g2), and TRAF (c110218_g1) were specifically down regulated in SP. Among these, FAR1, C2H2, GRAS, and TCP were closely related to pollen development.

At 48 h after pollination, growth-related hormones, such as auxin, cytokinin, and ABA^31^, were significantly downregulated, and after this time, the ABA levels increased and exceeded the normal level (Fig. 3A–C). At 48 h after pollination, the plant hormone signal transduction genes and proteins underwent corresponding changes; for example, auxin-related DEGs (7), zeatin-related DEG (1), and cytokinin-related DEPs (2) were significantly downregulated 48 h after SP (Supplementary Table S4).

#### RT-PCR validation

To determine the transcriptome’s reliability, 23 DEGs associated with “plant–pathogen interaction” (c126261_g1), “pyruvate metabolism” (c114981_g1), and “phenylpropanoid biosynthesis” (c72974_g2), among others, were selected (Supplementary Table S5). These selected DEGs with high repetitiveness verified the accuracy of the sequencing data (Supplementary Figure S4).

### DEPs in self- and outcrossed ovaries

**Figure 6:**
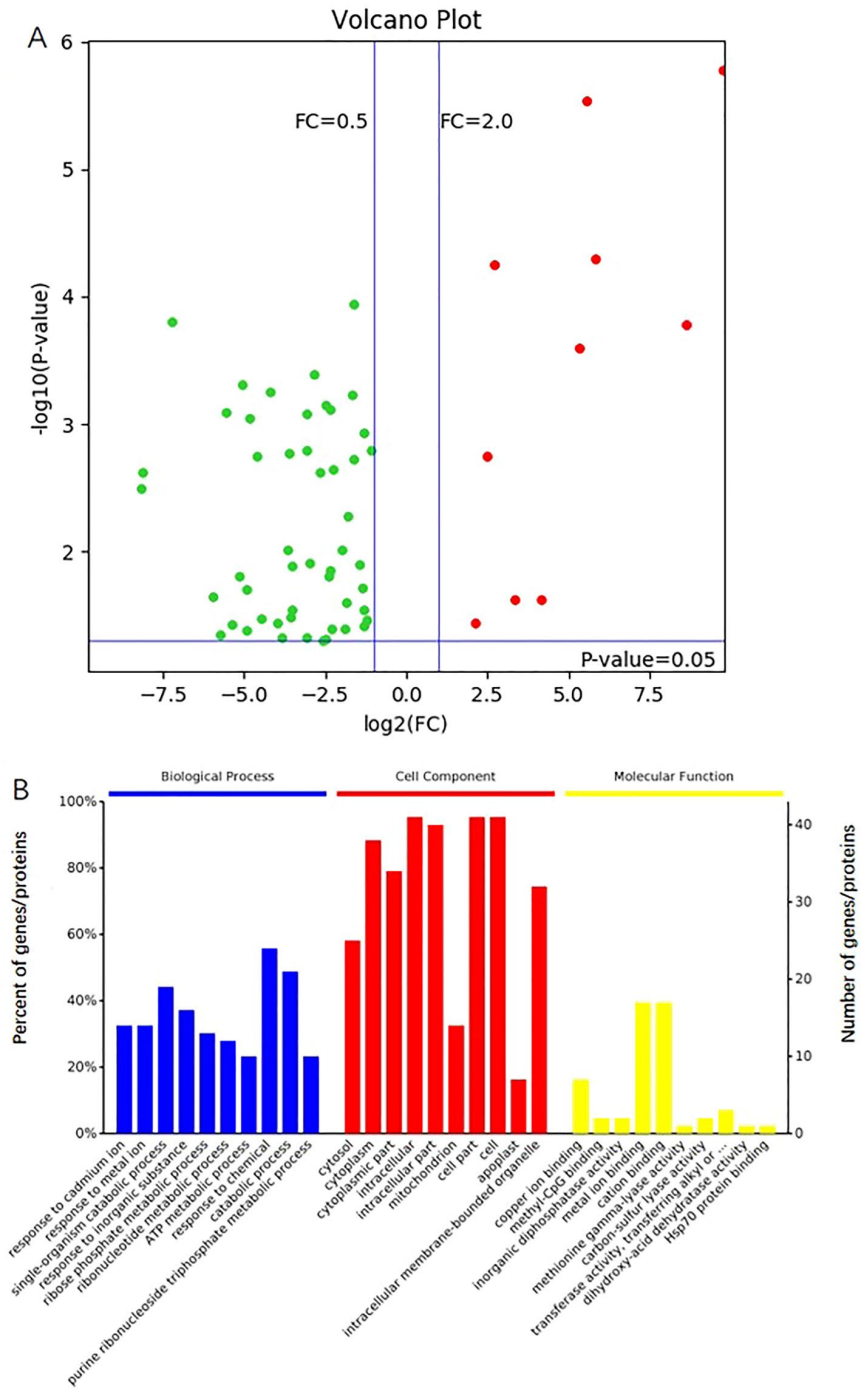
Volcano plot of DEPs (**A**) and GO term classification (**B**). A, Red plots indicate upregulation, and green plots indicate downregulation.

To further quantify gene expression in SP, protein expression pattern analysis was performed in SP versus OP ovaries at 48 h after pollination. DEPs were identified by a fold change ≥ 2.0 and ≤ 0.5, and *p* value ≤ 0.05 in S48 versus O48 (Fig. 6A, Supplementary Figure S5, and Supplementary Table S6). In total, 82 DEPs were identified, and more downregulated proteins (60) were observed in S48 (Fig. 6A). These DEPs were assigned to molecular functions (193 GO terms), cellular components (142 GO terms) and biological processes (786 GO terms), and there were 47, 94, and 229 significant terms, respectively (Fig. 6B, Supplementary Table S7). Most of these DEPs had ion binding, transferase, hydrolase, lyase, and oxidoreductase activity, and they were involved in the development, oxidation–reduction, organic substance metabolic, and cellular metabolic processes.

The categories were significantly upregulated in “oxidative phosphorylation” (COX6B-1|SS204; PDI|SS2022,SS7608; mitochondrial-like ATP synthase subunit d|SS2022, SS2015), and downregulated in “protein glutathionylation” (DHAR2|SS9106; GSTL2|SS2206), “glutamine biosynthesis” (GLNA|SS7412, SS8421), “aspartate family amino acid catabolic process” (MGL|SS8413; At5g55070|SS6511), and “oxidation–reduction process” (PER12|SS2509, SS2613; At1g75280|SS8315; GLX1|SS3305, SS4306, SS4307; At1g60710|SS8318; SDH1-1|SS8808; PDH2|SS4407; At5g55070|SS6511; At1g53240|SS8319; At3g02090|SS7706; CTIMC|SS8214) (Supplementary Table S7).

In total, 82 DEPs were identified using KEGG analysis and functional enrichment, and 30 pathways were enriched, 10 of which were significantly enriched in metabolism and biosynthesis (Supplementary Table S7).

### Association analysis between DEGs and DEPs

**Figure 7:**
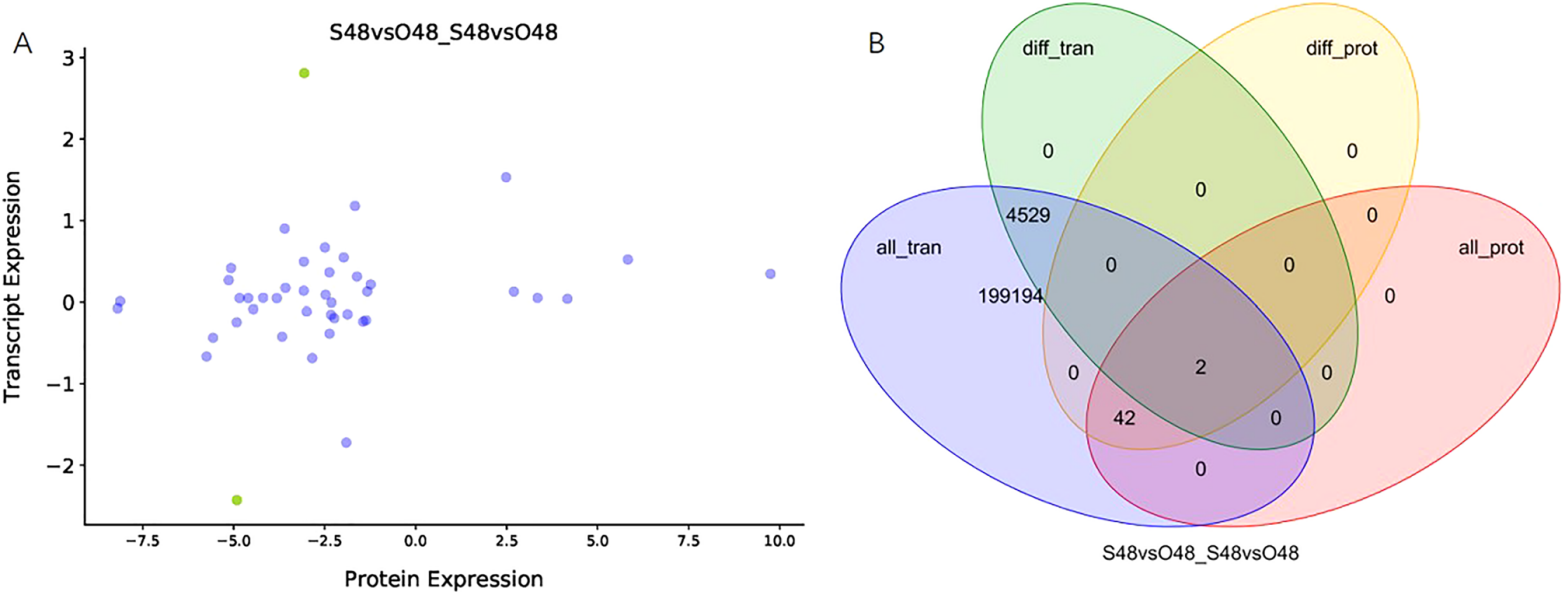
Association analysis between DEGs and DEPs. (**A**) Correlation scatter plot between DEGs and DEPs. The purple dots indicate the none significant difference protein, and the green dots indicate the significant difference protein. (**B**) Venn diagram showing the overlaps of RNA information obtained by transcriptome and protein information identified by the proteome.

Association analysis were performed to clarify the consistency of DEG and DEP expression patterns. DEPs did not have a significant correlation with DEGs at 48 h in the ovaries of SP versus OP (y = 0.047x + 0.1854, R^2^ = 0.0436, *P* = 0.179, Fig. 7A). Only c113206_g1| SS8413 and c104041_g1| SS2022 were co-detected from the DEP–DEG association analysis (Fig. 7B). Forty-one proteins and genes annotated in 3 GO functional enrichments were aggregated into four clusters, and only cluster 3 was upregulated in cells (Supplementary Figure S6A). A total of 24 proteins and genes categorized into 7 KEGG pathways were also aggregated into four clusters (Supplementary Figure S6B). These pathways were mainly in oxidation–reduction and basal metabolism.

## Discussion

SI is an important mechanism that protects flowering plants, allowing them to overcome inbreeding depression and providing a high level of heterozygosity^32^. Pollen tubes grow from the stigma to the ovaries, and penetrate the ovules. This forms a zygote and successfully promotes fruit and seed formation. A series of complex signaling controls then occurs. Incompatible pollen can be killed at any stage of this process. Based on our previous^30^ and present studies, the morphological, anatomical, and gene expression patterns have been comprehensively explored to unravel the complexity of LSI in *S. superba*. The time point of 48 h, as implicated in this study, was important for PT elongation to the ovary and penetration of the ovule. The transcriptome and proteome data showed that DEGs and DEPs were more enriched in the “oxidation–reduction process,” “ribonuclease T2 activity,” “cell wall or membrane biosynthetic progress,” and “transmembrane transport” in ovaries of SP relative to ovaries of OP, while “recognition of pollen,” “plant hormone signal transduction,” and “Glycolysis” were downregulated (Supplementary Figure S2 and Supplementary Table S8).

### Incompatible PT growth and its physiological changes

Researchers have already partly revealed the SI mechanism in some species in Theaceae^15,22,26,27,33^. Seth^27^ and Chen^33^ found that the PTs of SP and OP of *C. sinensis* entered the ovary at 48 h; however, the IPT recognition system was expressed in the style. Zhang^22^ showed that PT growth in the SP of *C. sinensis* was hindered at 24 h in the style. The PT of SP and OP of *C. oleifera* entered the ovary at 60 h, but the PT of SP failed to penetrate the ovule. Therefore, 24 h for the style’s SI or the ovary’s LSI were two features in Theaceae. In our previous research, we found that PT growth stopped and that the IPT recognition system occurred in the ovary 48 h after pollination (Fig. 2)^30^. From the phylogenetic position of Theaceae and the fossil of *Schima*, it was shown that the *Schima* possessed n = 18 symmetric karyotypes, had 5, not 3 locules, had no center axis, and confirmed that the Schimeae (≡Gordonieae) belonged to an ancient taxon and that this branch should be an early node on the phylogenetic tree in Theaceae^24,25,29,34,35^. We found the ovary’s LSI in *S. superba* was not effective; during self-pollination, 11.2% still developed fruit^30^, and *C. sinensis* had only 1.1%^27^. This may be for an IPT abortion in the ovary, and this distance may not have been enough to encounter more lethal factors than that from the style to the ovule.

Reactive oxygen species (ROS) are the reactive products of oxygen that have the potential to damage living cells, and they play a key role in diverse development stages, such as self-incompatibility during pollination to induce PCD in incompatible pollen^36^. In plants, enzymatic and non-enzymatic antioxidants (proline, carotenoids, alpha-tocopherol, glutathione, ascorbic acid, flavonoids, and carotenoids) act as ROS detoxicants. In our results, GST-related proteins, such as the lambda GSTs (GSTL| SS2206) and dehydroascorbate reductases (DHARs| SS9106), which belong to the outlying minor GST classes, lactoylglutathione lyase (GLX1|SS3305, SS4306, SS4307), glutathione (GSH| SS7412| SS8421| c112627_g1| c86003_g1| c90752_g1| c97760_g1), and SOD were all downregulated at 48 h in the ovary of SP. The reduction of detoxification factor levels in SP induces ROS accumulation and damage that can be lethal^35–38^.

### PT guidance and IPT recognition

PT guidance is divided into two processes: pre-ovular and ovular guidance, both of which occur in the ovary^37^. Receptor-like kinases (RLKs) mediate signaling pathways to control ovular guidance^38,39^. In our results, 16 G-type lectin S-receptor-like serine/threonine-protein kinases, which included 5 RLK1 (c126066_g2| c100047_g1| c100047_g3| c103929_g1| c126606_g1) and one RKS1 (c106281_g1), in addition to one PT reception gene at rapid alkalinization factor (RALF), c86998_g1, were significantly downregulated at 48 h in the SP ovary.

S-RNase, which is exclusively expressed in pistil, is associated with female determinants in GSI and implicated in genetically identical pollen and the rejection of self-pollen, and it would trigger mitochondrial collapse and IPT’s PCD^40^. In our study, three S-RNase genes, which contain the catalytic domain of T2-type ribonuclease (PF00445), c110815_g1, c115031_g1(RNS1) and c94788_g1(RNS3), were significantly upregulated at 48 h in the ovary of SP. Comparing these three genes with other S-RNases in Rosaceae, Plantaginaceae, Rutaceae, Solanaceae, and Rubiaceae (Supplementary Figure S7), c110815_g1 was found to have a close relationship with *Prunus* T2/S-type RNase. When comparing the deduced amino acid sequences of these three S-RNases with the S-RNases reported for *Prunus* species, we identified introns I, II, and III, five conserved regions, and a hypervariable region located between C2 and C3 (Supplementary Figure S8). The three S-RNases possessed the HVa (RHV), HVb, and RC4 regions, which are considered prime candidates for the *S* specificity-determining region, mainly in Rosaceous S-RNases^41^. Interestingly, c110815_g1 had three introns: the first was next to the *Prunus*-specific intron and located in C1; the second was located in the same position as in *Prunus* S-RNase in RHV; and the third was in RC4. We presumed that this gene could have a special function in self-incompatibility.

**Figure 8:**
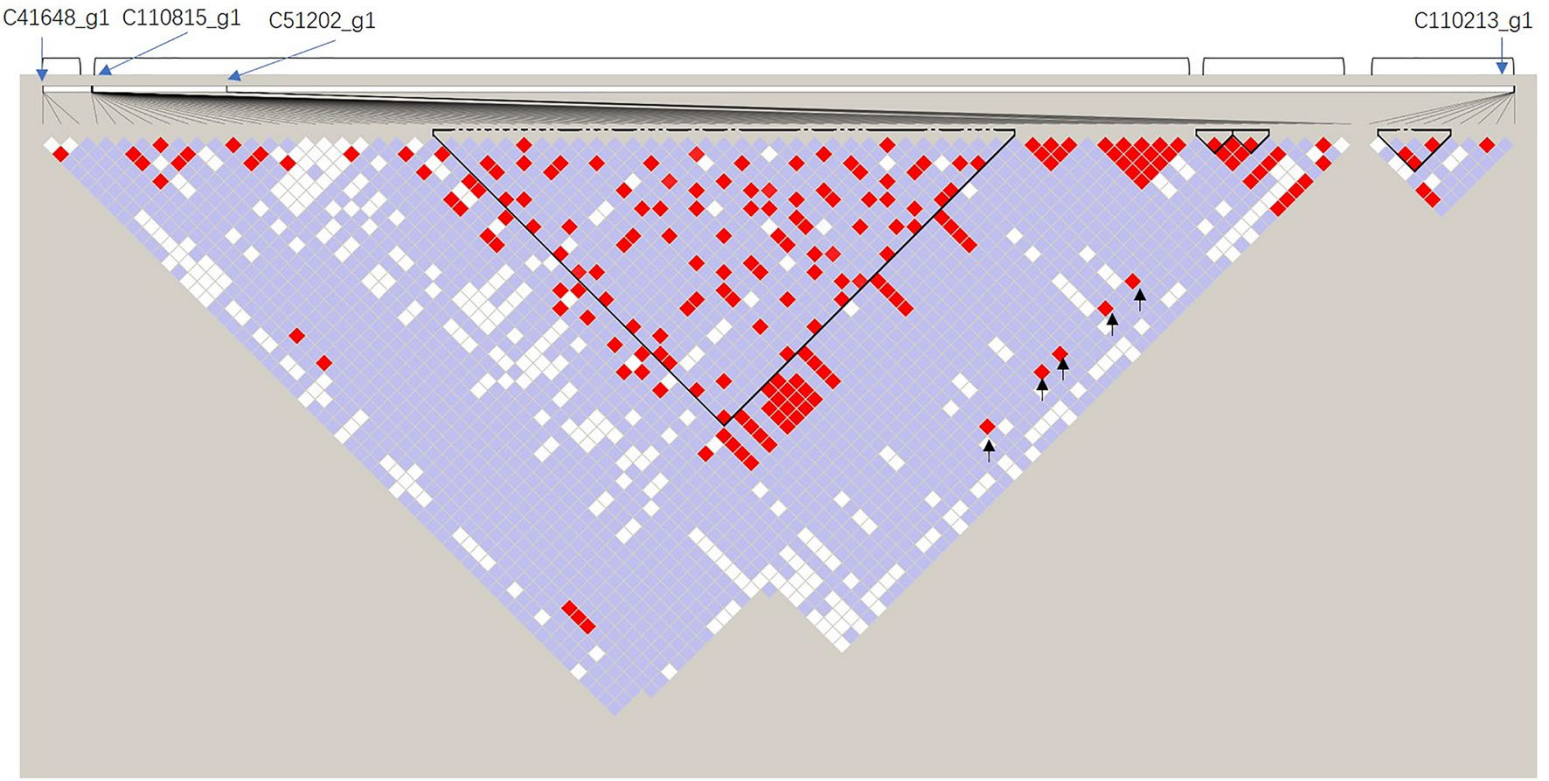
Linkage disequilibrium (LD) plot among C41648_g1, C110815_g1, C51202_g1 and C110213_g1. The color for each box in the LD plot represents the LD relationship, showing increasing LD from white to red.

**Figure 9:**
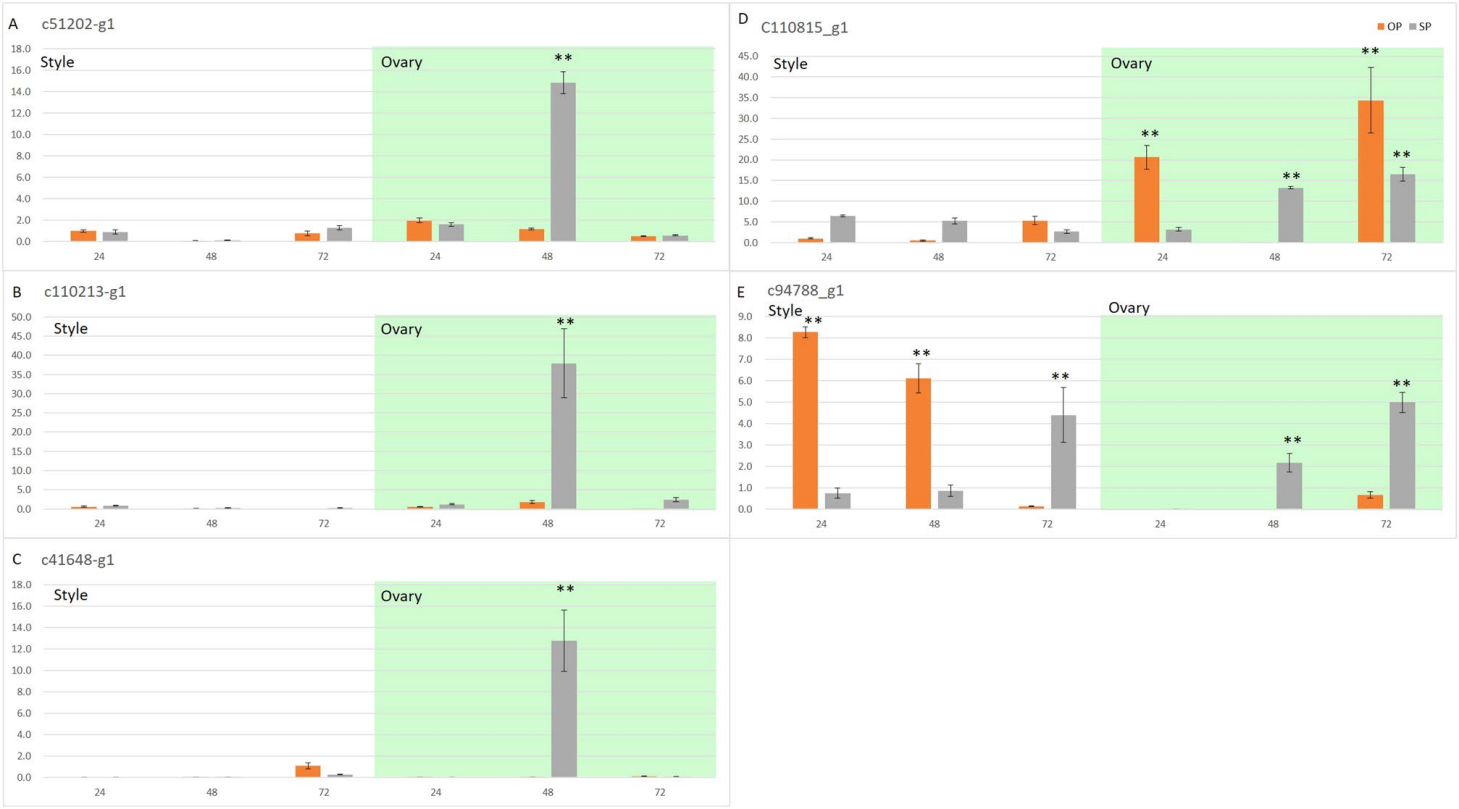
qRT-PCR expression analysis of 3 SFB genes (**A**–**C**) and 2 S-RNase (**D**,**E**) using GAPDH as the internal control from 24 to 72 h in the style and ovary of SP and OP. **p values (0.05).

The male determinant that interacts with S-RNase degradation is the S-locus region, which contains the novel F-box protein named SLF/SFBs^42^. The SLF/SFBs interacted with cognate S-RNase and prevented non-self S-RNase catalytic activity. When non-self S-RNase enters the PT, it forms an SCF complex and targets the S-RNase for ubiquitination and degradation; PT then continues growth. Self S-RNase binds to the recognition domain, not the active domain, resulting in dysfunction of SCF for polyubiquitination of self S-RNases. Then, self-S-RNases are released to trigger subsequent PT growth inhibition events^43^. In our results, 6 SFB putative genes were identified: F-box protein SKIP23-like (c88773_g1|c41648_g1) and F-box LRR-repeat protein (c120445_g3| c103038_g1| c51202_g1| c110213_g1). Phylogenetic analyses of *S. superba SFB*-like genes together with Rosaceae, Solanaceae and Plantaginaceae *SFB* and *SKP* genes, shown in Supplementary Figure S9, revealed that the c51202_g1 gene clusters with the *Prunus SFB* gene. Interestingly, c51202_g1 had 5 polymorphic SNP loci in the F-box domain, tightly linked to the RHV region (second intron) of c110815_g1 (Fig. 8). However, this gene was expressed in all tissues analyzed here (Supplementary Figure S10), whereas the S-pollen gene(s) were mainly expressed in anthers/pollen only, such as c41648_g1 and c110213_g1; thus, the c51202_g1 detected in *S. superba*, similar to *Prunus* species, may function as a general inhibitor (GI), as in *Prunus*, and c41648_g1 and c110213_g1 may function as SLF^40,44,45^. Further experiments should be conducted to clarify these proteins’ interactions and verify this presumption. qRT-PCR showed that c110815_g1 was mainly expressed in the ovary and increased in SP ovaries from 24 to 72 h, and c51202_g1, c41648_g1, and c110213_g1 were significantly upregulated at 48 h in the ovary of SP (Fig. 9). We presumed that these candidate genes were active in the self S-RNase’s cytotoxicity at 48 h in the ovary, and LSI in *S. superba* could be gametophyte controlled.

### Programmed cell death in IPT

Apoptosis or PCD is a highly conserved mechanism that removes unwanted cells in eukaryotes^46^. At 48 h in the ovary’s LSI in *S. superba*, the IPTs ceased growth and died. As mentioned above, the S-RNase induces apoptosis or PCD by direct degradation of ribosome RNA or indirect phosphorylation of the protein, destabilization of cytoskeletons, and release of cytochrome *c* (cyt *c*)^47,48^. Indirect progress related to Ca^2+^ signaling is transduced by protein phosphorylation, and this signal transduction mechanism relates to mitogen-activated protein kinase (MAPK) cascades. The Ca^2+^ concentration in the cytosol can also trigger downstream sensors to form stimulus-specific information. In *S. superba*, one MAPK gene c120765_g5 was significantly upregulated, and the “Ca^2+^ channel activity”, “calcium ion transmembrane transporter activity,” and “calcium ion transport” pathway-related genes and proteins were also upregulated.

The important prophase characteristic of PCD inhibits mitochondrial outer membrane permeabilization (MOMP), and the mitochondria system loses its function^49^. Then, some soluble proteins, such as cyt *c*, diffuse from the intermembrane space (IMS) to cytosol^50,51^. GO analysis of DEGs showed that 30 GO class 14 DEGs related to mitochondria, such as inner mitochondrial membrane protein complex, mitochondrial membrane organization, and mitochondrial proton-transporting ATP synthase complex, were downregulated, and none of these were upregulated at 48 h in the ovary of SP (Supplementary Table S9). This demonstrated that, at 48 h in the ovary of SP, the mitochondrial membrane system was dysfunctional, and PCD had already started. In addition, during apoptosis, Bcl-2 family proteins regulate soluble proteins (e.g., cyt *c*), which are released from mitochondria^48,52^. The Bcl-2-associated athanogene-like protein gene c122719_g2, which contained the BAG domain, was 2.5-fold more highly expressed at 48 h in the ovary of SP (Supplementary Table S10). Kang reported that *AtBAG6*, which contains the BAG domain, was overexpressed in Arabidopsis, and promoted mitochondrial fusion, causing cyt *c* release into the cytosol and inducing PCD^52–54^.

Cyt *c* leakage from the mitochondria into the cytosol is an initial marker for PCD^47,48^. In our study, the concentrations of cytochrome oxidase COX (SS204) and ATP synthase (SS2015) were 9.7- and 5.8-fold higher, respectively, at 48 h in the ovary of SP. COX is the enzyme that catalyzes the oxidation of reduced cyt *c* by molecular oxygen. A higher COX concentration at 48 h in the ovary of SP indicates that the plants have already started the self-protection mechanism due to higher levels of cyt *c*. If the COX concentration exceeds the critical value, the activity is limited by either the cyt *c* or ascorbate concentration, and PCD occurs^59^. These results are consistent with the results of the disordered oxidation–reduction system shown previously.

## Conclusions

In this study, the analysis of PT growth characteristics, internal physiology changes, transcriptome, and proteome of SP and OP ovaries revealed the LSI mechanism in *S. superba* and identified several candidate genes. The time point 48 h after pollination in the ovary’s LSI in *S. superba* was different from 24 h after pollination in style’s SI in *C. sinensis*. High expression levels of 2 S-RNase genes and 3 SLF-related genes suggest that LSI in *S. superba* is under gametophyte control. Self S-RNase induced PCD of IPT could occur by indirect transduction of Ca^2+^ signaling and release of cyt *c*. Mitochondrial fusion and function loss caused the PCD; in addition, oxidation–reduction system disorder promoted cell death. These results revealed the LSI molecular mechanism in *S. superba* and provided a reference for other plants in the Theaceae family.

## Methods

### Plant materials and pollination treatment

This study was conducted by the Forestry Genetic and Breeding Lab at the Research Institute of Subtropical Forestry, CAF, State Forestry Administration, China (RISF-CAF). The State Forestry Administration is responsible for national parks and other protected areas. No specific permission was required for these locations/activities, as they were based on a non-destructive collection of plant material. The species is not endangered or protected, and the locations are not privately owned or protected by law.

Two cultivars, ‘JO59’ and ‘YX1’, grown in Lanxi nursery, Zhejiang province, China, were used in this study. These cultivars were identified, bred, and preserved by our institution. The scions were collected from selected trees of the natural forest of *S. superba* from Jianou and Youxi in Fujian; they were then grafted on local stock and maintained in Lanxi until 2013. The collection of plant material complied with institutional, national, and international guidelines. Field studies were conducted in accordance with local legislation.

In May 2019, at 9:00–11:00 am on sunny days, we performed pollination [self-pollination (SP) was ‘JO59’ × ‘JO59’, outcross-pollination (OP) was ‘JO59’ × ‘YX1’]. The styles and ovaries were collected at different intervals (2, 4, 8, 12, 16, 24, 36, 48, 60, 72, 84, 96, and 120 h after pollination).

Then, the styles and ovaries of each sample with three replications were frozen in liquid nitrogen and stored at − 80 °C for enzyme activity, hormone, amino acid, protein, and RNA-seq analysis.

### Enzyme assays

Three defense-related enzymes, peroxidase (POD), catalase (CAT), and superoxide dismutase (SOD), were analyzed using SP and OP ovaries from 24 to 120 h. POD activity was determined using the guaiacol–catalase activity method^55^. SOD activity was measured using inhibition in the photoreduction of nitro blue tetrazolium (NBT)^56^. CAT activity was measured spectrophotometrically by monitoring the decrease in absorbance at 240 nm^57^.

### Hormone extraction and determination

Using HPLC–MS/MS, the levels of auxin (IAA), abscisic acid (ABA), zeatin (ZT) were determined by Zoonbio Biotechnology Co., Ltd. (Nanjing, China)^58^. The SP and OP ovaries (0.1 g fresh weight), with three biological replicates from 24 to 120 h, were selected to extract the hormone, and HPLC–MS/MS analysis was performed (Agilent 1290). HPLC analysis was carried out using a poroshell 120 SB-C18 column (2.1 mm × 150 mm; 2.7 μm). The parameters were set as follows: spray voltage, + 4500 V; automizing temperature, 400 °C; the pressure of the air curtain, nebulizer, and aux gas were 15, 65, and 70 psi, respectively. SAS v8.0 (SAS Institute, Cary, NC, USA) was used to analyze the data.

### Transcriptome analysis

The total RNA from the S48 and O48 ovaries was extracted using an RNAprep pure Plant Kit (Dingguo, Beijing, China). RNA quality and quantity were verified using 1% agarose gels and Agilent 2100 (Agilent Technologies, CA, USA). The mRNA was enriched and purified by magnetic beads with oligo T (dT), cleaved and synthesized first-strand cDNA using random hexamers. The double-strand cDNA libraries were then purified using the AMPure XP system (Beckman Coulter, Beverly, USA). Transcriptome sequencing was performed using the Illumina HiSeq™ 2000 sequencing platform in Novogene (Tianjin, China). The adaptor and low-quality sequences (Q < 20 or less than 35 bp) were removed in raw reads. Then the clean reads were assembled using Trinity assembler^59^. The functions of the unigenes were annotated using Blastx searches with 1e-5 against the protein databases. Blast2GO software^60^ was used for gene Ontology (GO) term analysis. The plant Transcription Factor Database (PlnTFDB) (version 3.0) was used to determine the transcription factors (TFs)^61^. FPKM was used to estimate the expression of each gene^62^, and the DESeq R package 1.10.1 was used to estimate the differential expression between the two treatments^63^. DEGs were determined with an adjusted *p* value < 0.05 determined by DESeq. The R-seq data were uploaded to the sequence read archive (accession no. SUB6596208).

### Protein extraction and expression analysis

With some modification, protein extraction was performed following Isaacson^64^. The SP and OP ovaries at 48 h were frozen and ground to power, and 1 g of the sample was used for protein extraction^65^. Then, 10 μg samples were run on 12% SDS-PAGE gel and visualized by CBB stain^66^. Gel images with 300 dots per inch were scanned using an image scanner (GE Healthcare, USA). A total of 450 μl solution with 1500 μg protein sample was used to finish isoelectric focusing with the following parameters: 50uA per strip, rehydration at 50 V for 8 h, 100 V for 1 h, 200 V for 1 h, 500 V for 1 h, 1000 V for 1 h, 1000–10,000 V (gradient) for 1 h, 10,000 V for 13 h, 500 V for 12 h, temperature, 20 °C. The Ettan-DALT-Six system was run for 45 min at 100 V and then at 300 V for 6–8 h. Using PDquest 8.0 software, all gel images were processed in three steps: spot detection, volumetric quantification, and matching. The differential protein spots were selected using two thresholds (*p* ≤ 0.05, fold change ≥ 2 or ≤ 0.5). An ABI 5800 MALDI-TOF/TOF Plus mass spectrometer (Applied Biosystems, Foster City, USA) was used for peptide MS and MS/MS detection, and CalMix5 was used to calibrate the instrument (ABI5800 Calibration Mixture). GPS Explorer V3.6 software (Applied Biosystems, USA) with default parameters was used for data integration, and the proteins were identified using the MASCOT V2.3 search engine (Matrix Science Ltd., London, U.K.).

### Transcriptome and proteome association analysis

The corresponding transcripts were identified using the gene ID of the proteome, and the corresponding relationship between the protein and the transcript was determined. A series of association analyses were then perfomed. Correlation analysis was conducted on the fold change (taken as log2) of genes (proteins) identified by transcriptome and proteome analyses in the two omics stydies. The drawing process was implemented using Python. The different multiples of the corresponding transcriptome genes were determined in the two omics studies. The difference multiples were taken as log2 and then plotted using the ComplexHeatmap of the R package. GO annotation of the transcriptome and proteome was used for GO function enrichment using the tool wego (http://wego.genomics.org.cn/). Clustering heat map analysis of GO/KEGG function enrichment was conducted using the fold change (taken as log2) of DEPs identified using the proteome and corresponding transcriptome genes. The drawing process was implemented using ComplexHeatmap.

### RT-PCR

Using specific primers designed by Beacon Designer 7.90 (Premier Biosoft International, Palo Alto, CA, Supplementary Table S5), the 22 expressed transcripts were validated. The candidate gene expression patterns were tested in two ways: (1) tests in organs to determine whether they were expressed in whole plants (leaves) or pollen (fresh and obtained from stamens before pollination), style, or ovary; (2) candidate genes were also detected in the style and ovary (contained the pollen tubes) of SP and OP at 0, 24, 48, and 72 h to clarify the trend in the gene expression pattern and which time point was important. To synthesize cDNA, 100 ng of total RNA in the ovaries was used (PrimeScript™ RT reagent Kit, Takara, Dalian, China). Then, RT-PCR was performed on the ABI Quantstudio 7 Flex Real-Time PCR System (Applied Biosystems) using a SYBR® Fast qPCR Mix (Takara, Dalian, China). The program was as follows: 95 °C for 30 s, 40 cycles at 95 °C for 15 s, and 60 °C for 30 s. The reference gene was set as *GADPH*. Three biological and technical replicates were performed and the gene expression relative quantitation was calculated using the 2^−ΔΔCt^ method^67^.

### Ethics approval and consent to participate

All field studies were performed in accordance with the local legislation in China and complied with the convention on trade in endangered species.

## Supplementary Information

Supplementary Figures.

Supplementary Tables.

## Data Availability

The RNA-seq and proteome datasets in this article are available in supplementary files. All raw data were submitted to the NCBI Sequence Read Archive: BioProject ID PRJNA625576.

## Abbreviations

LSI: Late-acting self-incompatibility
PCD: Programmed cell death
HR: Hypersensitive response
SI: Self-incompatibility
SSI: Sporophytic SI
GSI: Gametophytic SI
OSI: Ovarian SI
SP: Self-pollen
OP: Outcross-pollen
DEGs: Differentially expressed genes
DEPs: Differentially expressed proteins
PT: Pollen tube
IPT: Incompatible PT
FAA: Formalin-Aceto-Alcohol
Lpt: PT length
Ls: Total style length
KEGG: Kyoto Encyclopedia of Genes and Genomes
GO: Gene ontology
TFs: Transcription factors
POD: Peroxidase
CAT: Catalase
SOD: Superoxide dismutase
NBT: Nitro blue tetrazolium
IAA: Indole-3-acetic acid
ABA: Abscisic acid
ZT: Zeatin
HPLC: High-performance liquid chromatography
CC: Cellular component
BP: Biological process
MF: Molecular function
COX: Cytochrome *c* oxidase
LRR-RLKs: Leucine rich repeat receptor like kinase
RALFs: Rapid alkalinization factors
SRK: S-locus receptor kinase
SCR: S-locus cysteine-rich protein
SCF: SKPl-Cullin1-Rbx1-F-box
S-RNase: S-locus-encoded ribonuclease
MAPK: Mitogen activated protein kinase
cyt *c*: Cytochrome *c*
IMS: Intermembrane space
*Bcl*: B-cell lymphoma
DHAR: Dehydroascorbate reductase
GSH: Ascorbate–glutathione
GST: Glutathione S-transferase
GSSG: Oxidized glutathione
APAF: Apoptotic protease activating factor
