# Supplementary material for: Incompatible pollen abortion and late-acting self-incompatibility in Schima superba
Source: Sci Rep. 2022 Sep 16;12:15598. doi: 10.1038/s41598-022-19946-3 (PMC9481619; doi:10.1038/s41598-022-19946-3)
Supplement: Supplementary file 1 — Supplementary Figures. [file 41598_2022_19946_MOESM1_ESM.pdf]

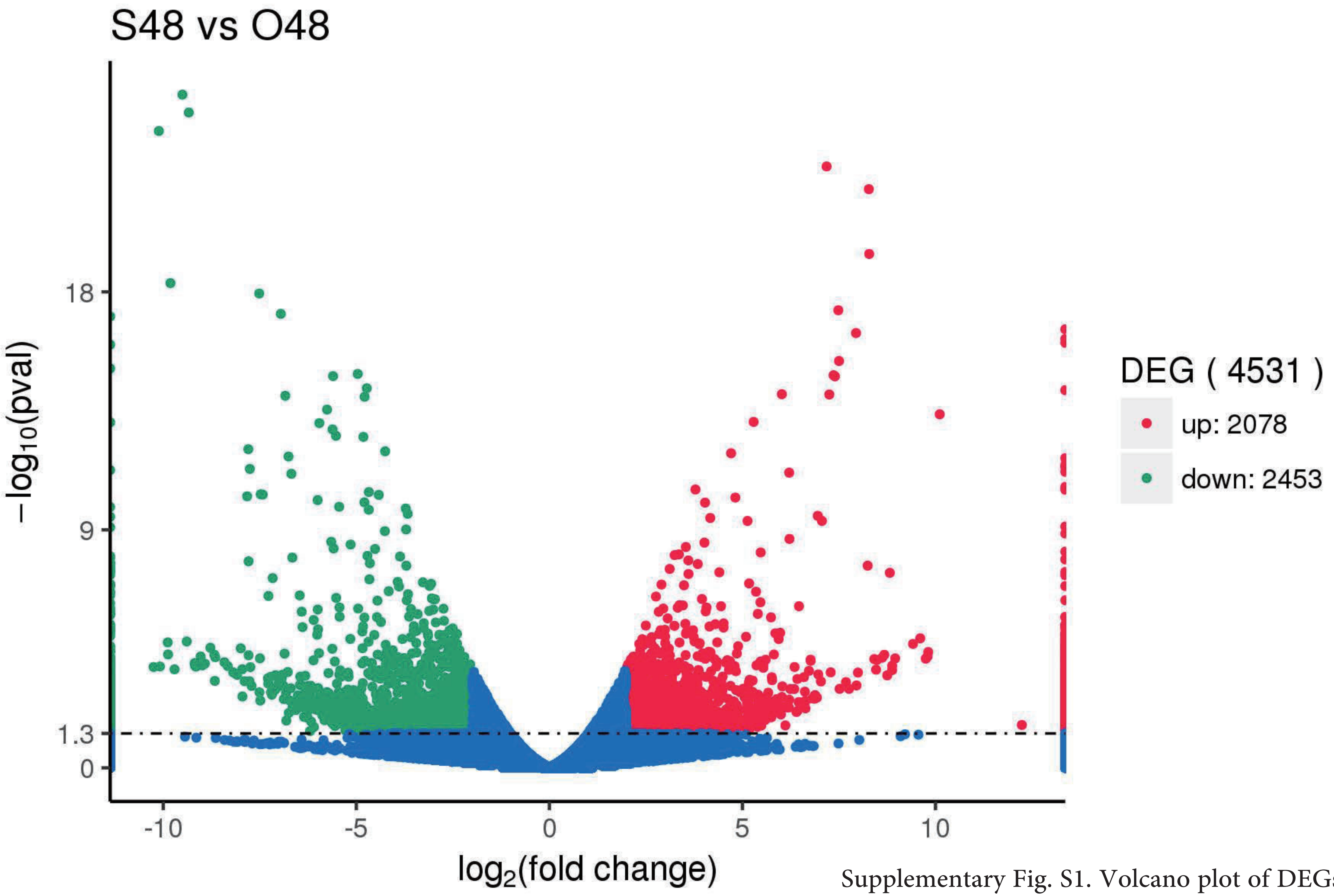

Supplementary Fig. S1. Volcano plot of DEGs.

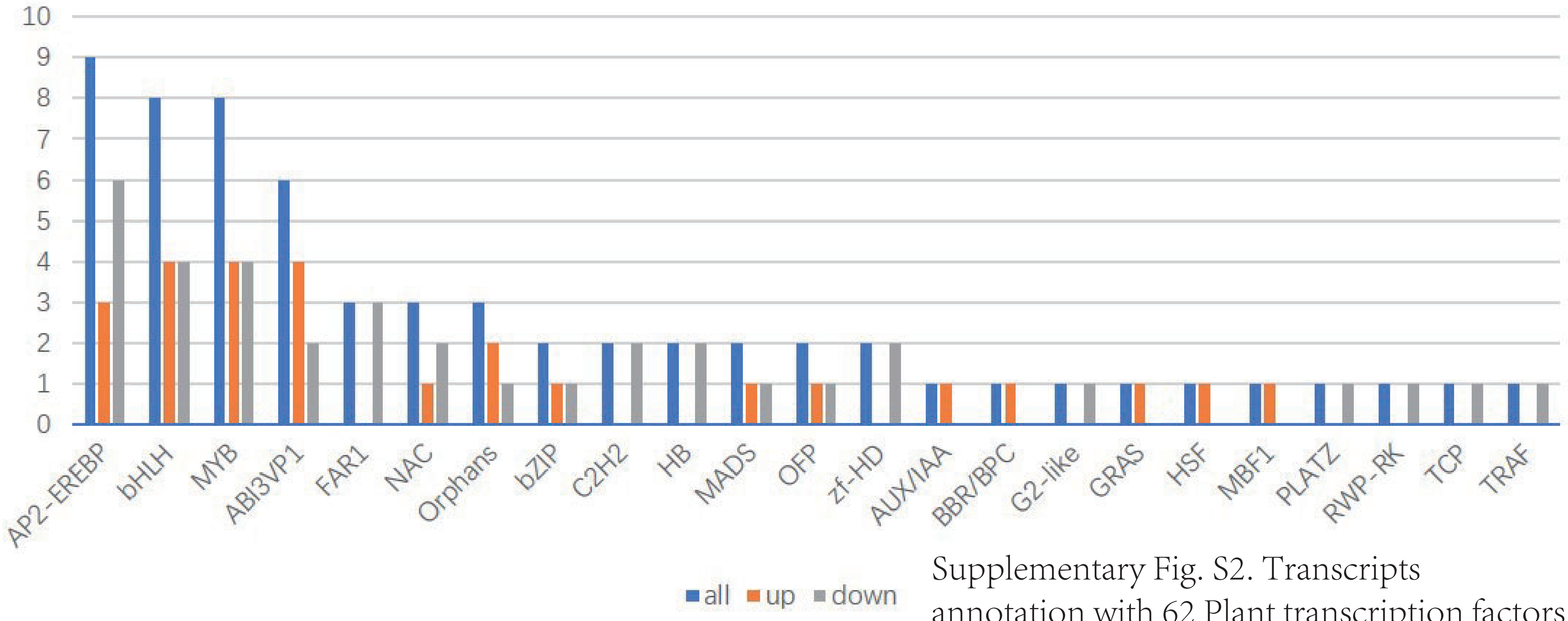

Supplementary Fig. S2. Transcripts annotation with 62 Plant transcription factors.

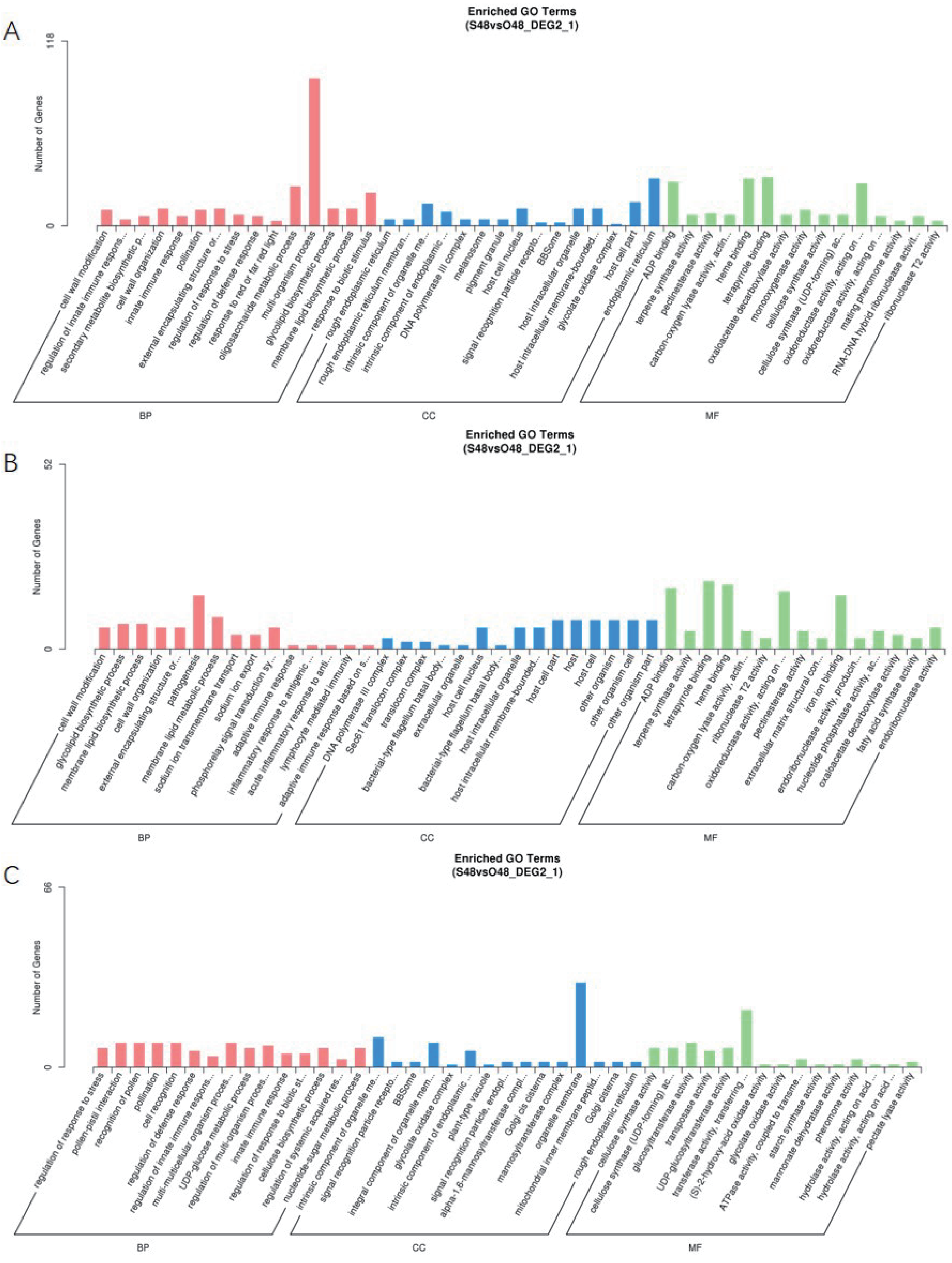

Supplementary Fig. S3. Top 15 GO enrichment analysis of DEGs of all, upregulated and down regulated transcripts in S48 vs O48.

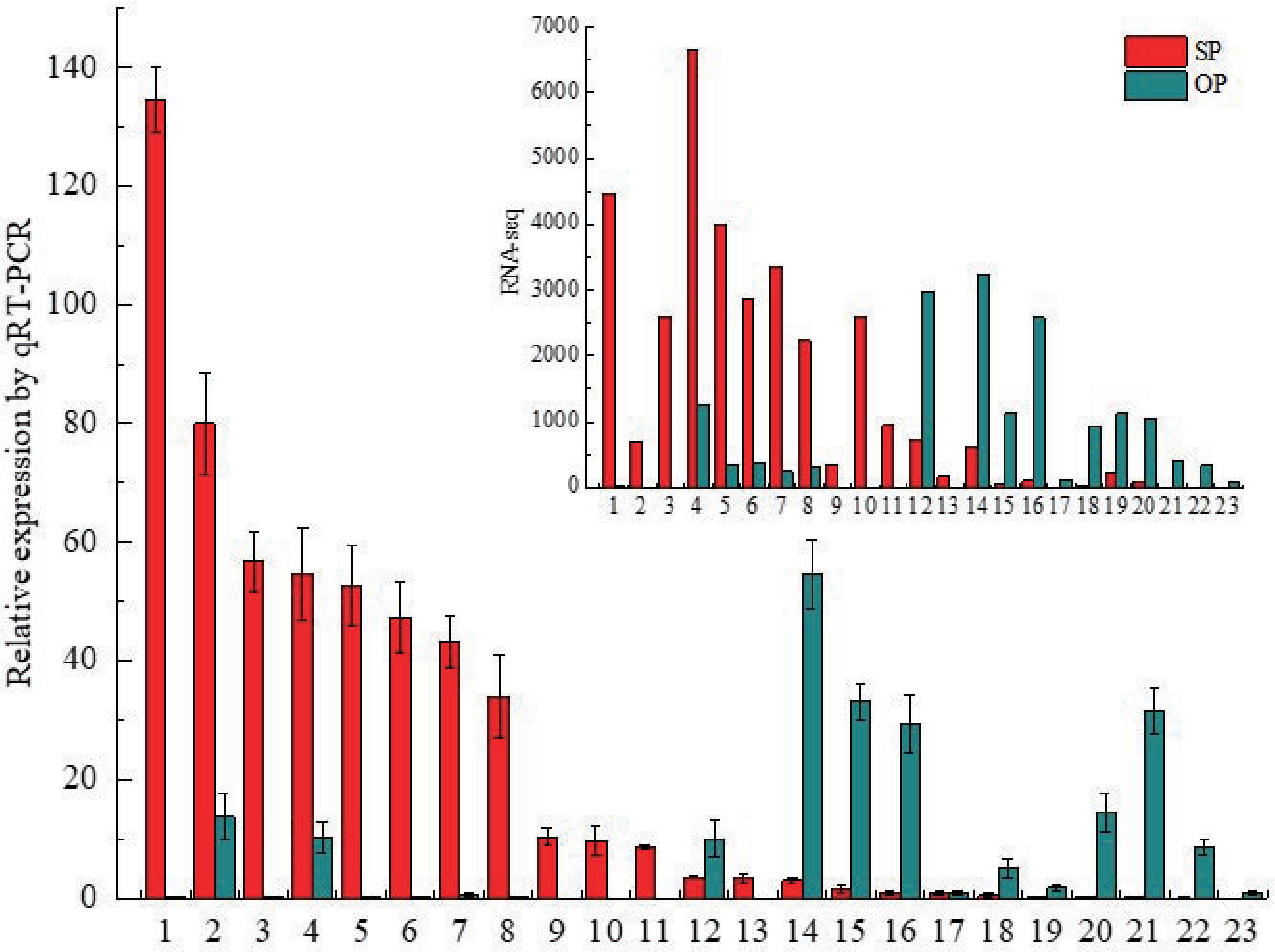

Supplementary Fig. S4. Validation of RNA-seq results by qRT-PCR.

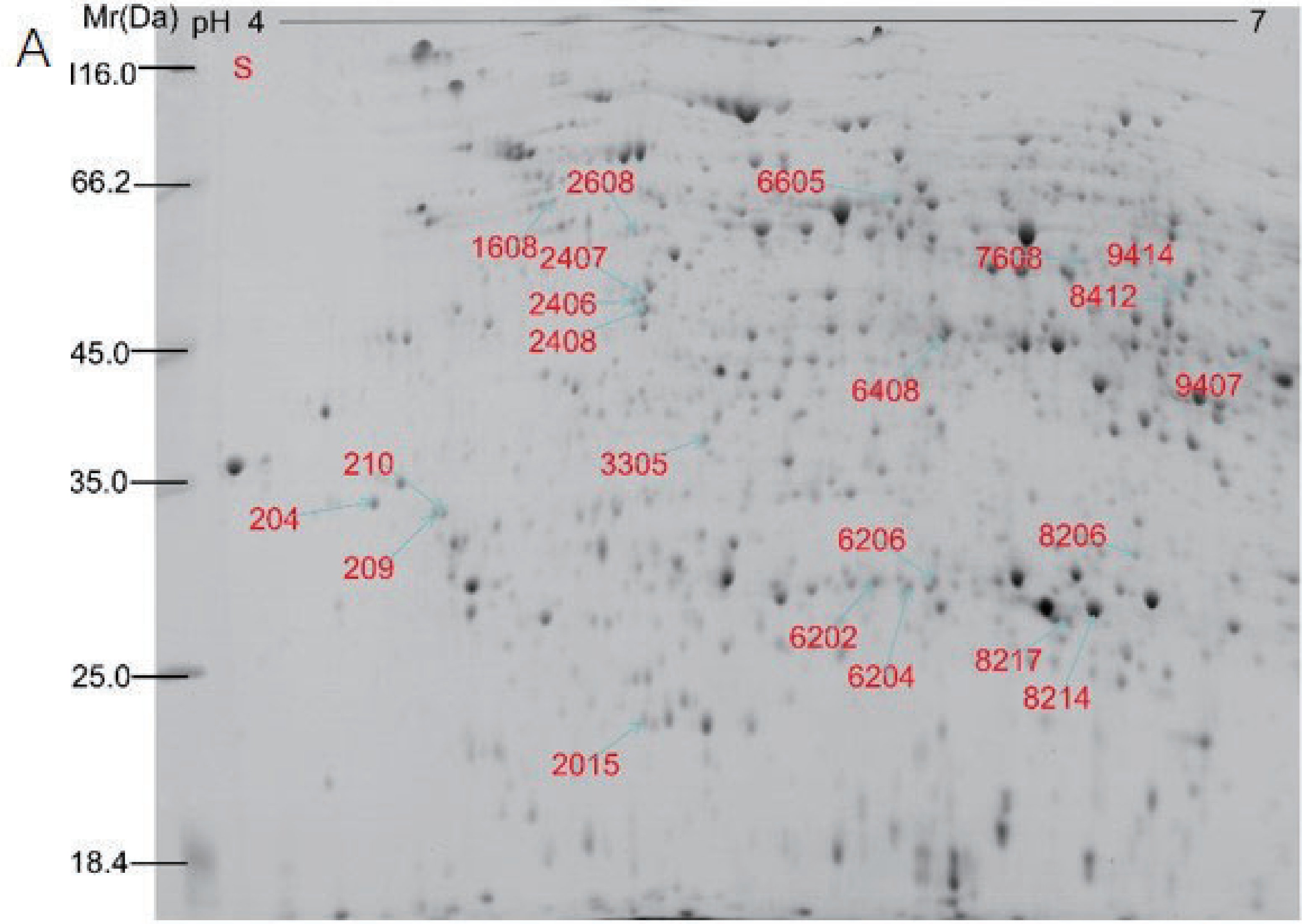

Supplementary Fig. S5. The 2D ovary protein profile of SP (A) and OP (B).

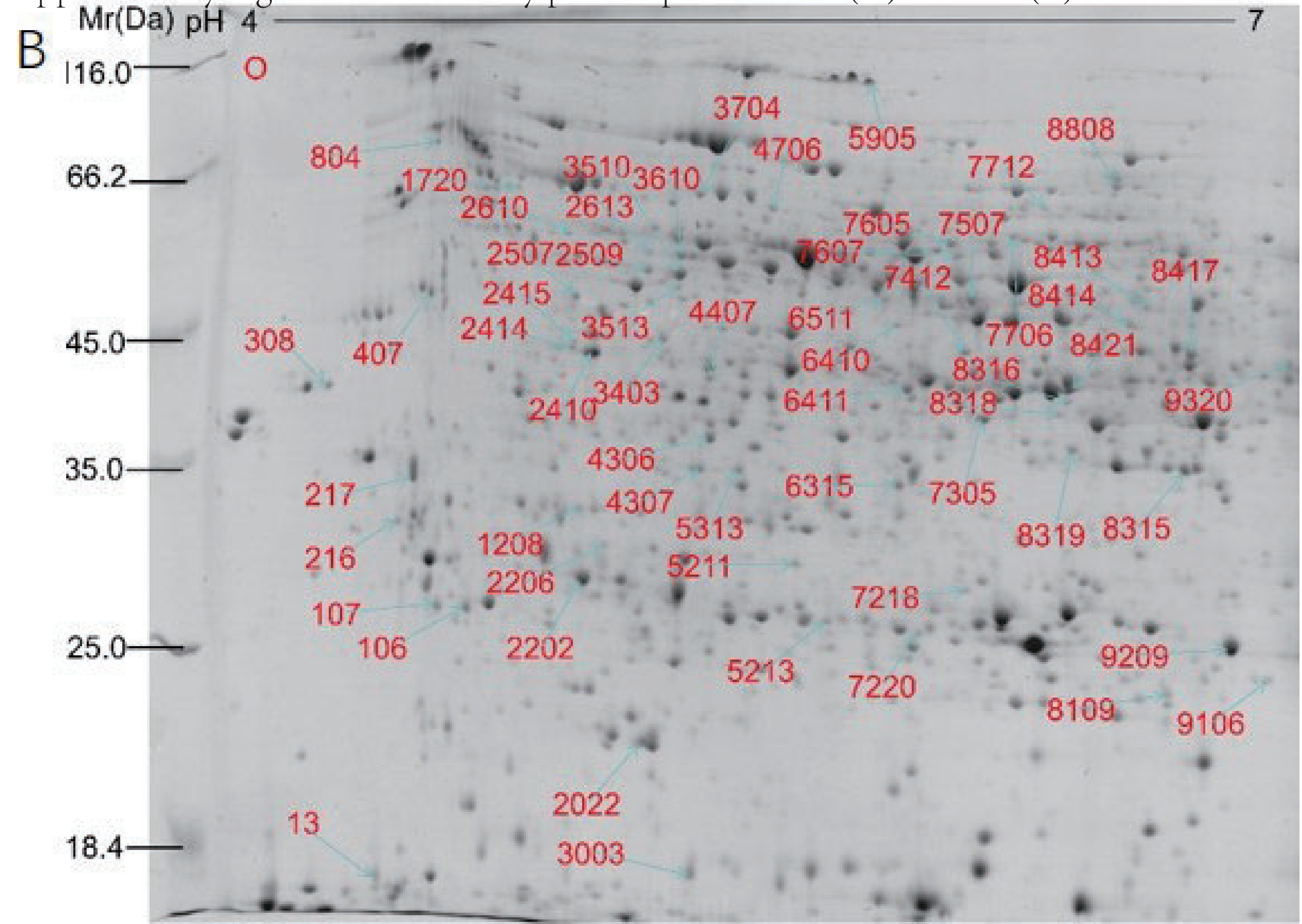

Supplementary Fig. S6. Expression patterns of clustered proteins/genes in association analysis in GO (A) and KEGG (B) pathway enrichment.

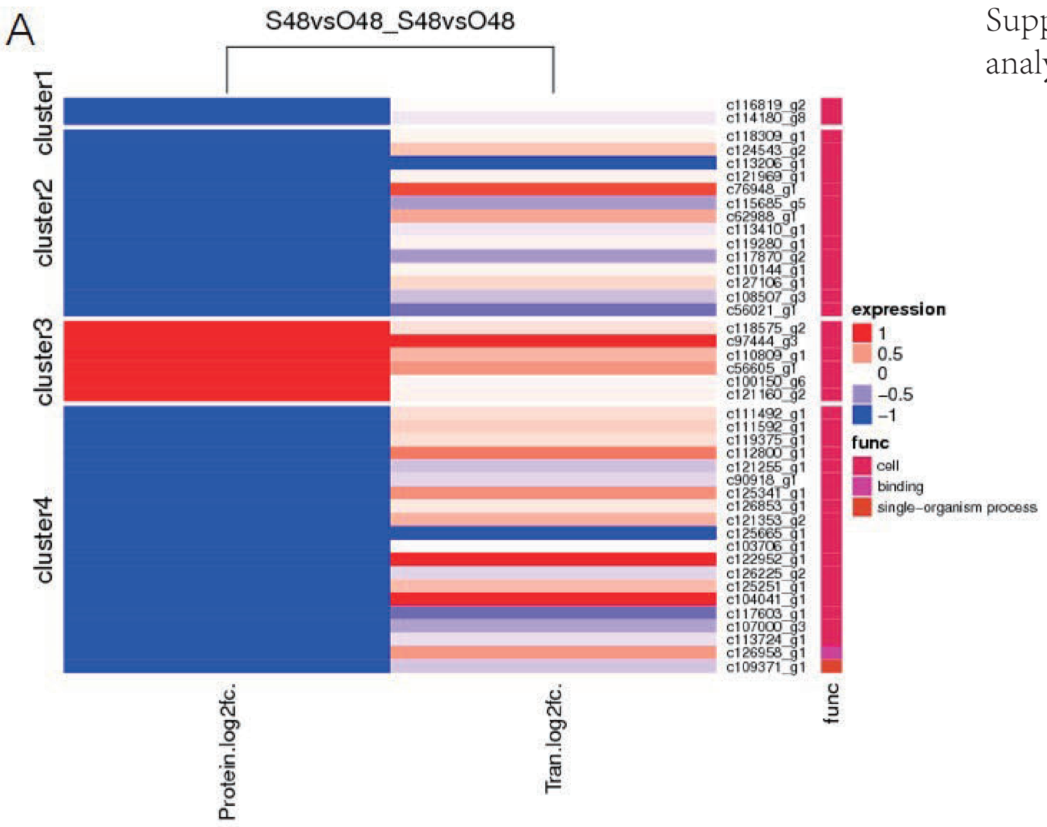

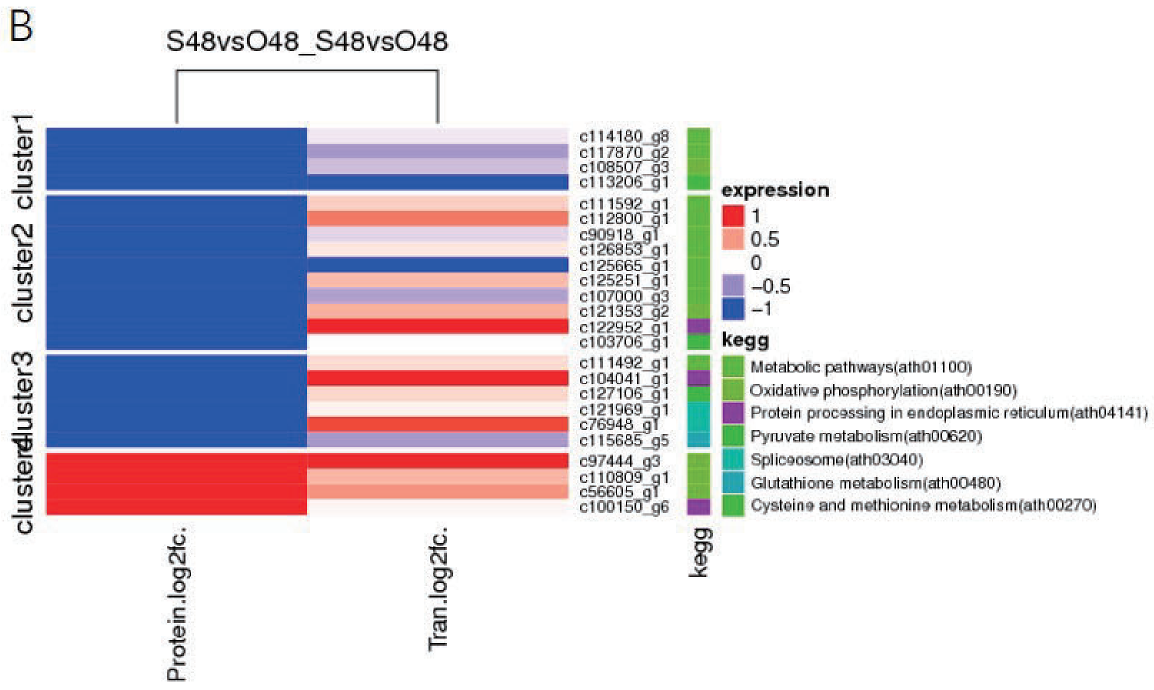

Supplementary Fig. S7. Neighbor-joining tree constructed using aligned amino acid sequences corresponding to the S-RNase genes from different taxa.

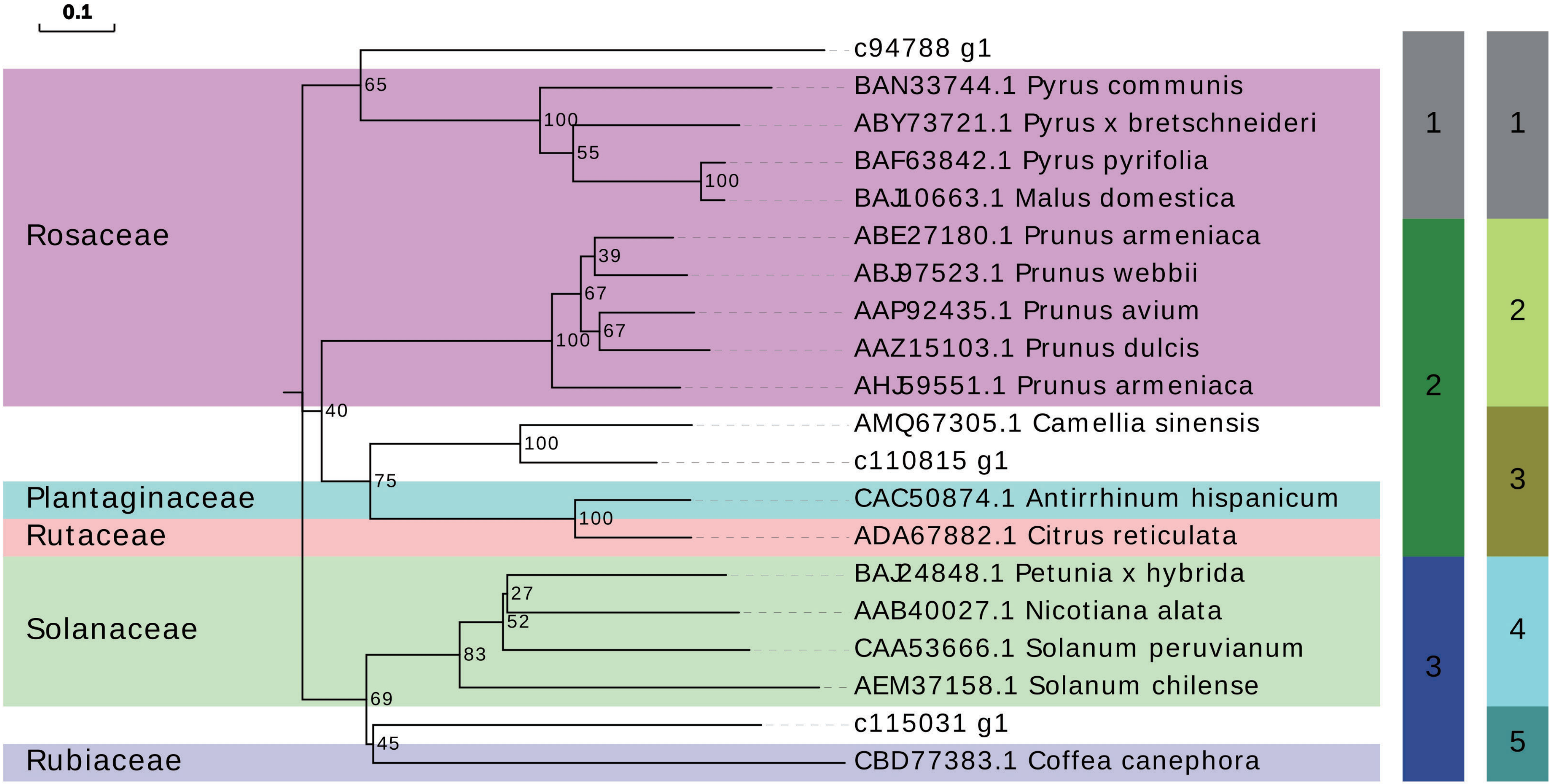

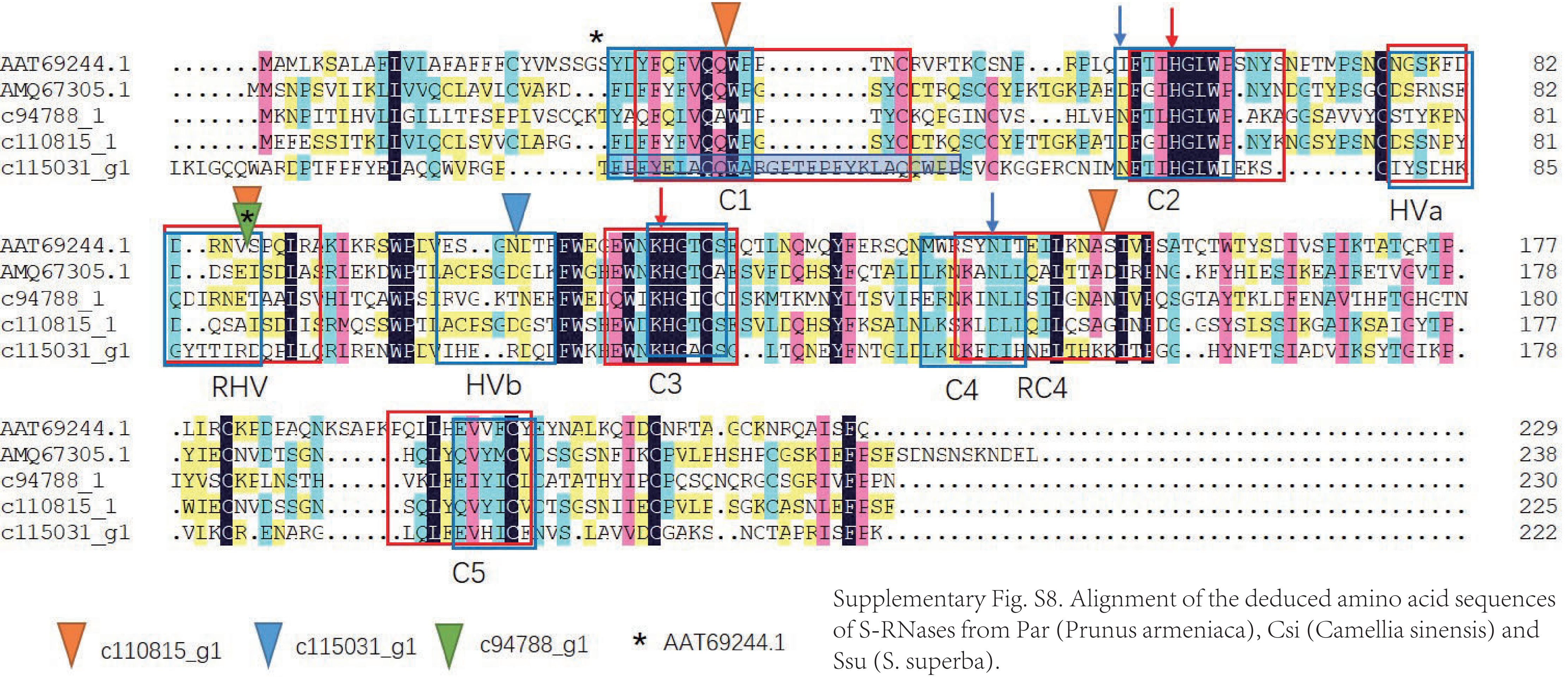

Supplementary Fig. S8. Alignment of the deduced amino acid sequences of S-RNases from Par (Prunus armeniaca), Csi (Camellia sinensis) and Ssu (S. superba).

Supplementary Fig. S9. Neighbor-joining tree constructed using aligned amino acid sequences corresponding to the S locus-linked F-box genes from different taxa.

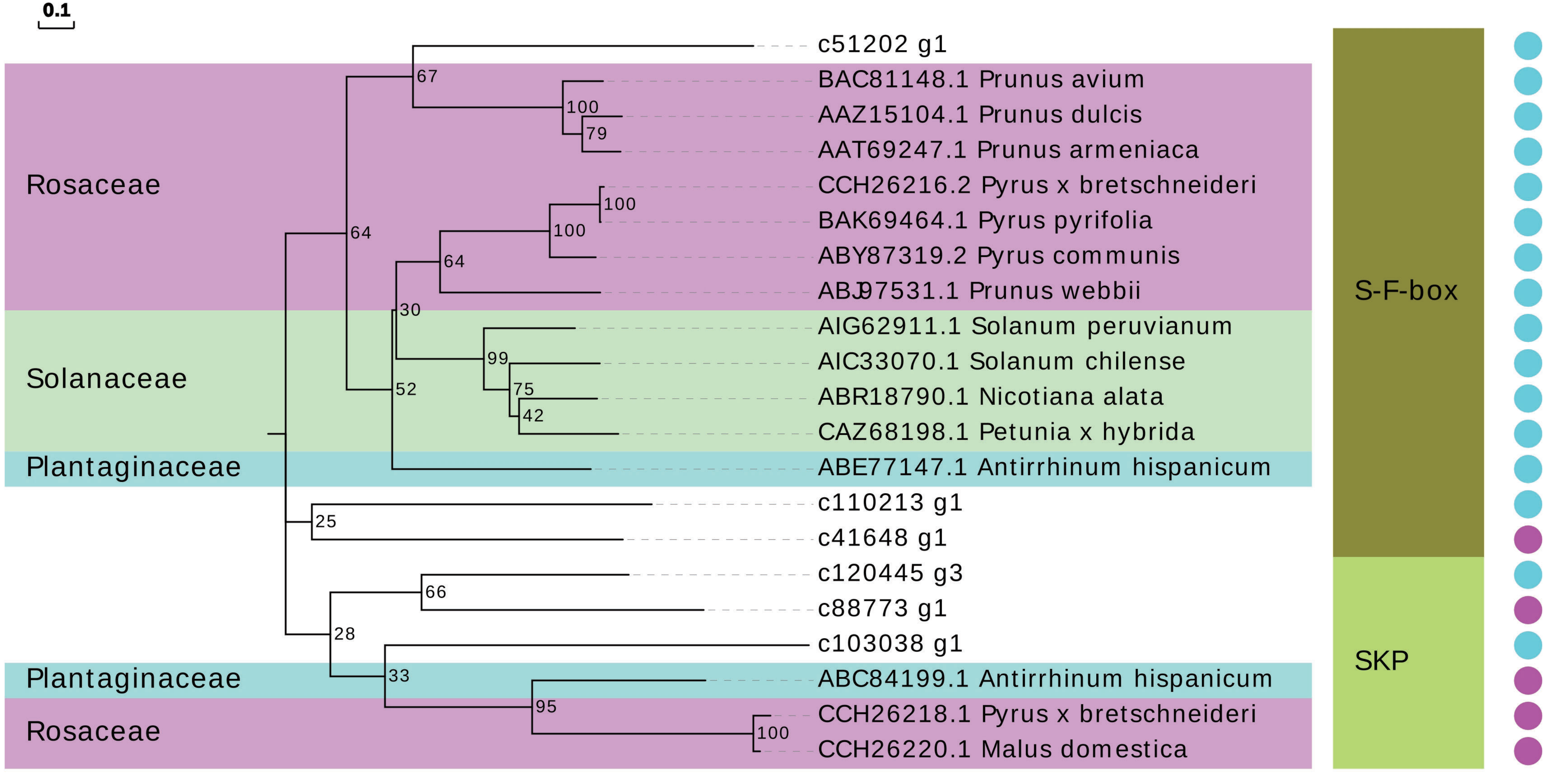

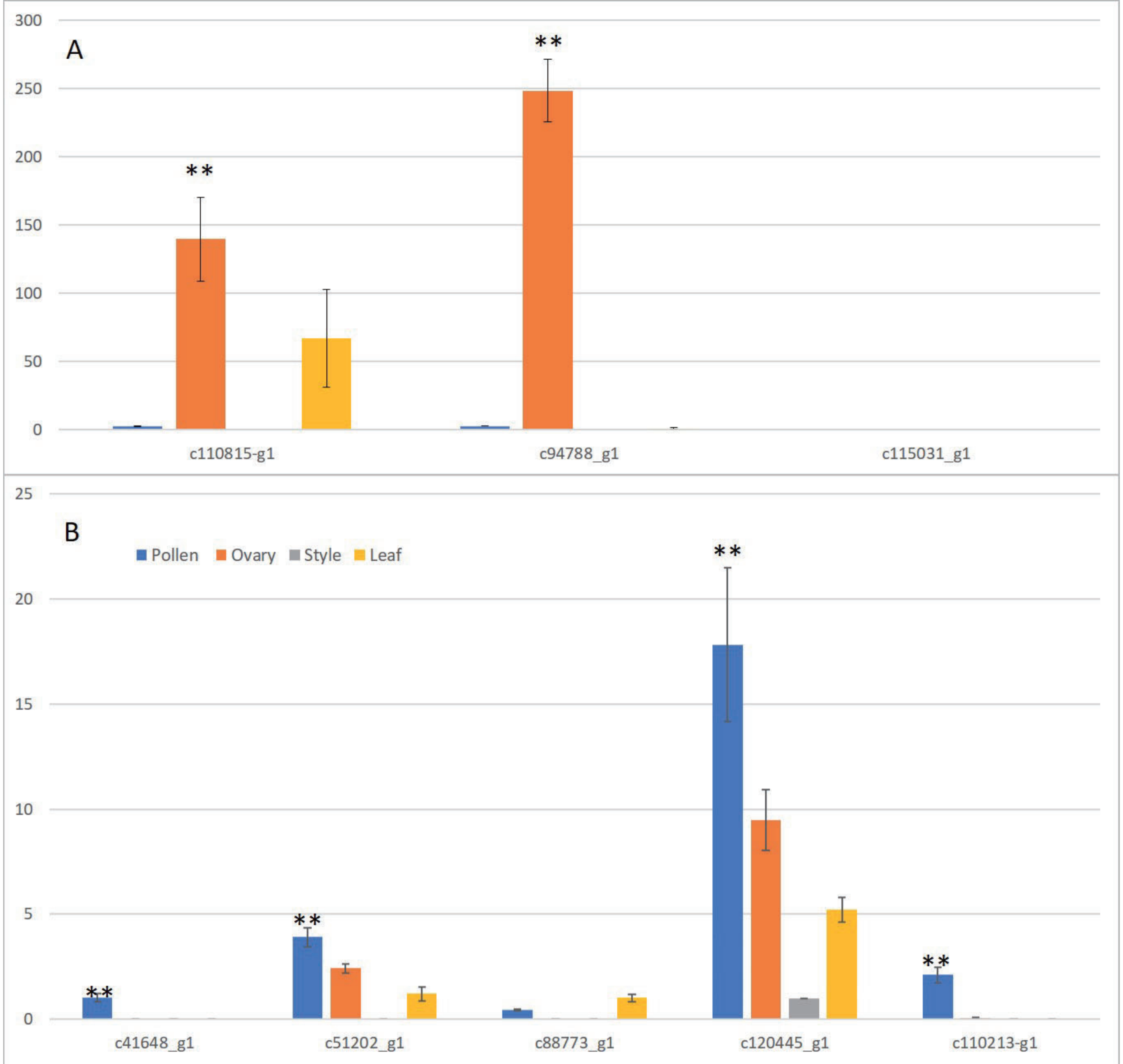

Supplementary Fig. S10. qRT-PCR expression analysis of 3 S-RNase (A) and 5 SFB genes (B).
